# Inhibitory effect of non-alcoholic compounds from spontaneously fermented beverage on *Helicobacter pylori*

**DOI:** 10.3389/fcimb.2026.1742545

**Published:** 2026-04-24

**Authors:** Cheng Fang, Ziyi Lei, Yehui Han, Pinghua Tang, Guanghui Yu, Jinyuan Sun, Bowen Wang, Yan Xu

**Affiliations:** 1 Laboratory of Brewing Microbiology and Applied Enzymology, Key Laboratory of Industrial Biotechnology of Ministry of Education, School of Biotechnology, Jiangnan University, Wuxi, Jiangsu, China; 2 Institute of Renhuai Jiangxiang Baijiu, Renhuai, Guizhou, China; 3 Key Laboratory of Geriatric Nutrition and Health (Beijing Technology and Business University), Ministry of Education, School of Food and Health, School of Light Industry Science and Engineering, Beijing Technology and Business University, Beijing, China

**Keywords:** Chinese Baijiu, gut microbiota, *Helicobacter pylori*, lactic acid (LA), non-alcoholic compounds

## Abstract

Global public health has long been threatened by *H. pylori* infection associated diseases. Spontaneously fermented beverage contains significant quantities of antibacterial and anti-inflammatory compounds. Here in this study, we demonstrated that the non-alcoholic compounds (NACs) of Baijiu, a traditional spontaneous fermented alcoholic beverage of China, have significant *H. pylori* inhibitory activity. It can ameliorate *H. pylori* infection-induced inflammation both *in vitro* and *in vivo*. Furthermore, NACs intervention reverses *H. pylori* infection-induced alteration of gut microbiota, especially boosting colonization of the beneficial gut commensal *Lactobacillus*, *Akkermansia*, *Eisenbergiella*, *Ruminococss*, and *Bifidobacterium*. Prediction of gut microbiota function indicated that NACs reversed the increase in a series of metabolic pathways induced by *H. pylori* infection, including those associated with Alzheimer’s disease, pathways in cancer etc. The non-targeted metabolomic analysis reveals 384 compounds in NAC, including 142 organic acids. Amongst these compounds, the content of lactic acid is as high as 1.26 g/L. Lactic acid at such concentration effectively inhibited the growth of *H. pylori*, reduced urease activity and transcript levels virulence genes (VacA, CagA), and decreased *H. pylori* infection-induced increase of cytokines (IL-6, IL-1β) in GES-1 cells. Our work proposes that Baijiu NAC could serve as a candidate for the supportive eradication of *H. pylori*. More importantly, it expands upon the existing limited knowledge of the impact of *H. pylori* infection on gut microbiota.

## 1 Introduction

*H. pylori* infection has a global prevalence in excess of 50% (Hooi et al., 2017). A series of adverse gastrointestinal outcomes are linked to *H. pylori* infection. These outcomes include chronic gastritis, gastric ulcers and cancer (Wizenty and Sigal, 2025). Epidemiological data showed that approximately 90% of adenocarcinoma are attributed to *H. pylori* infection (Plummer et al., 2015). Accordingly, *H. pylori* were classified as a Class 1 carcinogen by the World Health Organization (Møller et al., 1995). Notwithstanding the reduction in *H. pylori* prevalence in developed countries, attributable to changing hygienic standards and antibiotic treatments, it remains one of the most widespread bacterial infections on a global scale (FitzGerald and Smith, 2021).

*H. pylori* is a microaerophilic, spiral rod-shaped, gram-negative bacterium. It infects the epithelial lining of the stomach (Hooi et al., 2017). Mechanically, *H. pylori* manipulates the inflammatory response by translocating virulence proteins, namely cytotoxin-associated gene A (CagA) and vacuolating cytotoxin A (VacA) (Wizenty and Sigal, 2025). CagA is transferred through a type IV secretion system, and exerts a plethora of physiological effects that drive gastritis, ulcer and malignant transformation. VacA is secreted through the type V secretion system. Following internalization into epithelial cells via endocytosis, it induces vacuole formation, proinflammatory signaling and cell death. In recent years, the significance of the gastrointestinal microbiota in *H. pylori* infection-related diseases is increasingly recognized (Guo et al., 2020; Wizenty and Sigal, 2025). *H. pylori* infection modifies the local microenvironment by secreting urease which converts urea into ammonia and carbamate (Ansari and Yamaoka, 2017). This results in alterations to the composition of gastric microbial community (Chen et al., 2021). Furthermore, accumulating evidence indicate that *H. pylori* alters the structure of distant gut microbiota (Engelsberger et al., 2024), which is crucial for regulating both innate and adaptive immune systems (Honda and Littman, 2016). Nevertheless, whether specific bioactive compounds from food sources can protect against or alleviate *H. pylori* infection by reversing the infection-induced dysbiosis in the distal gut microbiota remains largely unexplored.

The current guidelines for treating *H. pylori* recommend the classic bismuth quadruple therapy (Liu et al., 2021). However, in tandem with the considerable utilization of antibiotics in agricultural and clinical contexts in the past few decades, the proliferation of drug-resistant *H. pylori*, coupled with the paucity of alternative antibiotic treatments, is resulting in significant challenges in the implementation of eradication therapy (Gisbert, 2005). Moreover, antibiotic use frequently engenders adverse reactions, such as diarrhea, which can diminish treatment compliance, and thereby exacerbating the global escalation in *H. pylori* resistance (Savoldi et al., 2018; Liou et al., 2019). The quest for milder alternative treatments has garnered mounting attention.

Alcoholic beverages are significant cultural and economic products with widespread consumption. However, the World Health Organization and a growing body of high-quality epidemiological evidence have confirmed that there is no safe level of alcohol consumption, and excessive alcohol intake can induce multiple systemic diseases including liver injury, cardiovascular disorders, and multiple types of cancer (Rumgay et al., 2021). Notably, previous epidemiological studies have reported contradictory findings regarding the association between alcoholic beverage consumption and *H. pylori* infection. While excessive alcohol intake disrupts the gastric mucosal barrier and exacerbates *H. pylori*-induced gastritis (Zhang et al., 2010), some population-based studies have observed that regular moderate alcohol consumption may be associated with facilitated *H. pylori* eradication (Murray et al., 2002; Kuepper-Nybelen et al., 2005; Du et al., 2023). Particularly, red wine can inhibit urease activity and prevent *H. pylori*-induced gastritis (Ruggiero et al., 2007; Paulo et al., 2011). Chinese Baijiu is a traditional spontaneous solid-state fermented alcoholic beverage, typically produced from sorghum as the main raw material, with wheat-based Daqu as the fermentation starter (Xu et al., 2022). The spontaneous solid-state fermentation process is responsible for the production of a significant number of bioactive non-alcoholic compounds (NACs), including organic acids, polyphenols and peptides. These compounds have been reported to possess excellent antibacterial and anti-inflammatory activity (Gao et al., 2025). Furthermore, our preceding studies observed the non-alcoholic compounds (NACs) in CB has the capacity to regulate gut microbiota structure (Fang et al., 2019b, 2022b, 2025). Therefore, it was hypothesized that the NACs have the potential to mitigate the inflammation and gut dysbiosis induced by *H. pylori*.

In this research, we observed that NACs showed significant anti-*H. pylori* effects both *in vitro* and *in vivo*. Mechanistically, NACs modulate the gut microbiota structure and potential functions by enhancing the growth of beneficial gut commensal bacteria. Further compositional analysis coupled validation experiments indicated that lactic acid contributed to the anti-*H. pylori* potential of NACs. To our knowledge, this study represents the inaugural investigation to document the inhibitory effect of NACs derived from spontaneously fermented alcoholic beverage on *H. pylori*.

## 2 Materials and methods

### 2.1 Baijiu sample, bacterial strains and cells

The Chinese Baijiu (CB) sample was collected from Renhuai, Guizhou Province. The alcohol concentration of is 53% vol. The non-alcoholic compounds (NAC) of CB were obtained by vacuum freeze drying. Specifically, 1000 mL of the CB sample yielded approximately 1.32 g of NACs. *H. pylori* ATCC 43504 were purchased from Zhili Zhongte (Wuhan) Biotechnology Co., Ltd. *H. pylori* was first cultured on Columbia blood agar (Qingdao Hope Bio-Technology Co., Ltd., China) under microaerophilic conditions at 37 °C for 48 h to obtain single colonies. Single colonies were then inoculated into Brucella broth (Qingdao Hope Bio-Technology Co., Ltd., China) supplemented with 7% fetal bovine serum (Wuhan PunoSai Life Technology Co., Ltd., China), and cultured under the same microaerophilic conditions at 37 °C for 48 h to obtain the bacterial suspension for subsequent experiments. Human gastric mucosal epithelial cell line (GES-1) was obtained from the Wuhan Shangen Biotechnology Co., Ltd. (China) and grown in RPMI-1640 (Gibco, USA), containing 10% fetal bovine serum, in controlled environment kept at 37 °C and 5% CO_2_.

### 2.2 The *in vitro* antibacterial activities

The antibacterial activities of CB and NACs were evaluated through the determination of the minimum inhibitory concentration (MIC). To this end, the stock solution of NACs or CB was serially diluted 2-fold with Brucella broth in a 96-well plate, to obtain a final concentration gradient ranging from 0.078 to 10 mg/mL. Following this, 10 μL of *H. pylori* solution (with a working concentration of 1 × 10^6^ CFU/mL) was added to each well. A well containing only medium acted as the negative control. The plates were subsequently incubated at 37 °C for 72 h. The lowest concentration of the drug in the well where no bacterial growth was defined as the MIC.

### 2.3 Cell culture and treatment

Following a 48-hour culture period, *H*. *pylori* were collected and suspended in RPMI-1640. The resulting concentration of *H*. *pylori* was determined to be 3 × 10^8^ CFU/mL by the plate colony counting method. GES-1 cells, at approximately 50% confluence (3 × 10^5^ cells), were then cultured in six-well plates for subsequent infection and treatment. In the absence of penicillin-streptomycin, the cells were categorized into four experimental groups: Control (Con), *H. pylori* (HP), Hp-CB (1% ethanol concentration), and Hp-NACs groups. The concentration of NACs is 24.9 mg/L, which corresponds to the concentration of lactic acid found in CB of 1% ethanol concentration. For the HP group, *H. pylori* were co-cultured with GES-1 cells at an MOI of 100:1 in a cell incubator for a duration of 24 h. In the Hp-CB and Hp-NACs groups, GES-1 cells were also exposed to *H. pylori* at the same MOI of 100:1 for 24 h, then treatment with CB or NACs for a further 24 h. The urease activity was detected using urease detection reagent (Beijing Suo Laibao Technology Co., Ltd, China), following the guidelines of the manufacturer.

### 2.4 Assessment of cell viability

The CCK-8 assay was utilized to assess cell viability. GES-1 cells, during their logarithmic growth phase, plated at a density of 4 × 10^4^ cells per well in 96-well plates and allowed to incubate for a duration of 24 h. Then, a series of RPMI-1640-diluted CB samples or ethanol (ethanol concentration were 0.5%, 0.8%, 1%, 2%, 3%, 4%, 8%, 10% v/v) were added. After another incubation period of 24 h, 10 μL of CCK-8 solution was dispensed to each well, and an additional incubation was conducted. A Thermo microplate reader was used to measure the absorbance at 450 nm.

### 2.5 RNA extraction and quantitative real-time PCR

Total RNA extraction was carried out utilizing the FastPure Cell/Tissue Total RNA Isolation Kit (Vazyme, China). Subsequently, the RNA underwent reverse transcription to synthesize cDNA through a commercial reagent kit (HiScript II RT SuperMix for qPCR, Vazyme, China), adhering to the instructions provided by the manufacturer. Amplification of the target gene was performed with the ChamQ Universal SYBR qPCR Master Mix (Vazyme, China). The qRT-PCR analysis was executed on an Applied Biosystems StepOne Real-Time PCR System (USA). The amplification protocol included an initial denaturation step of 30 seconds at 95 °C, succeeded by 40 cycles consisting of 10 seconds at 95 °C and 30 seconds at 60 °C, concluding with a 10-minute extension at 72 °C. The quantification of expression levels involved normalizing the Ct values of each gene against that of GAPDH, employing the 2^−ΔΔCt^ method, with assays carried out in triplicate. Genes used for PCR were enumerated in **Supplementary Table 1**.

### 2.6 Animal experiment

**Figure 1:**
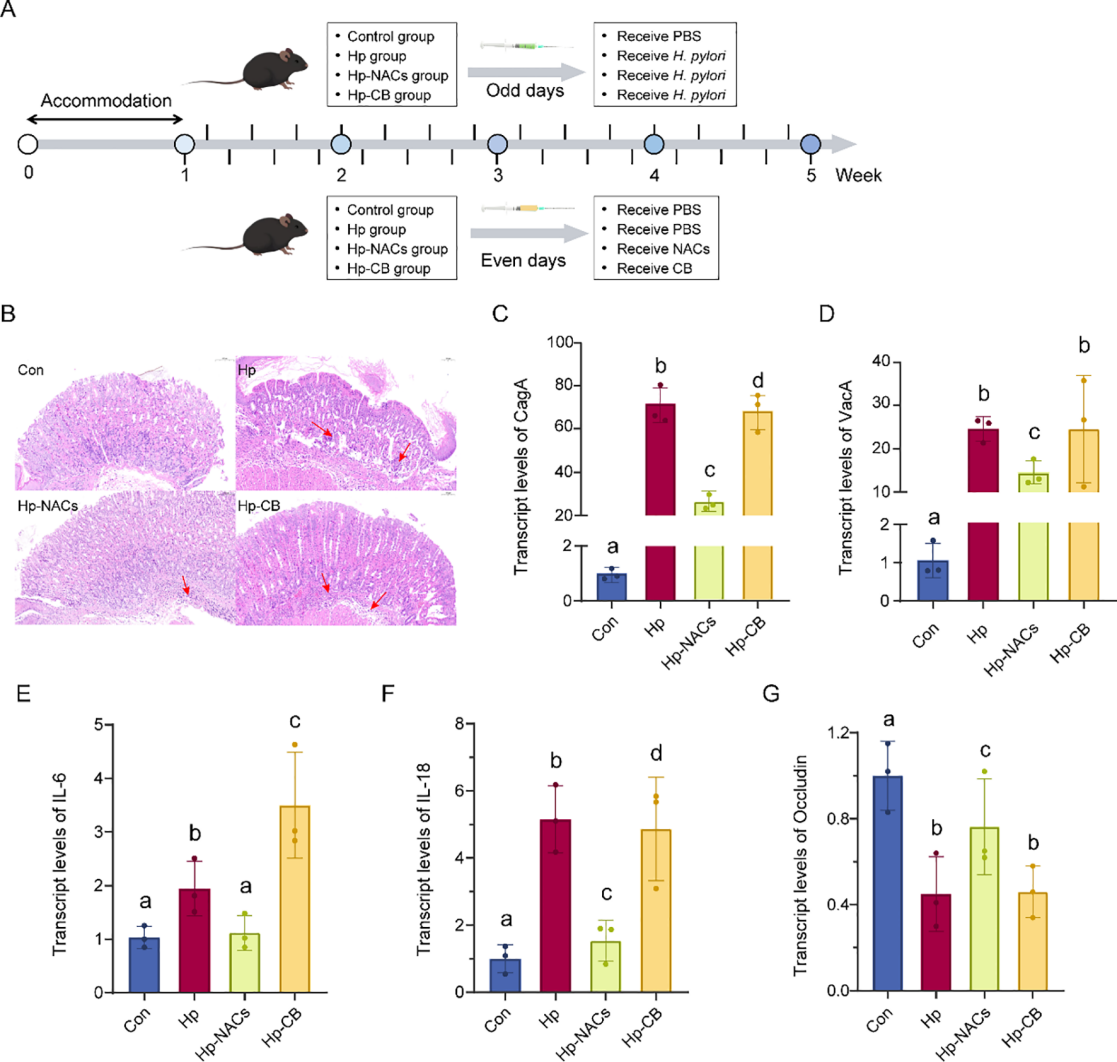
*In vivo* inhibition of NACs on *H. pylori*. **(A)** Overview of the mouse experiment and grouping flowchart. **(B)** Histological images representing H&E staining of the gastro. Original magnification, ×200. **(C)** Transcript levels of CagA. **(D)** Transcript levels of VacA. **(E)** Transcript levels of IL-6. **(F)** Transcript levels of IL-18. **(G)** Transcript levels of Occludin. Different lowercase letters above the bars indicate a statistically significant difference between groups (one-way ANOVA, P < 0.05); bars sharing the same lowercase letter indicate no significant difference between the corresponding groups.

The animal studies received approval from the Jiangnan University Animal Welfare and Ethics Committee. A total of 32 wild-type C57BL/6 male mice (20–22 g), which are specific pathogen-free (SPF), were obtained from SLAC Laboratory Animal Company located in Shanghai, China. These mice were kept in compliance with applicable regulations and had continuous access to water as well as a standard diet. The environmental temperature was controlled at 23 ± 2 °C. The relative humidity was maintained at 50 ± 10%. A light/dark cycle of 12 hours was implemented. After a week-long acclimatization phase, the 32 mice were randomly divided into four groups: (1) control group (Con), (2) *H. pylori* infection group (Hp), (3) *H. pylori* infection with Baijiu intervention group (Hp-CB), and (4) *H. pylori* infection with NACs intervention group (Hp-NACs). The arrangement and flowchart of the mouse experiment are illustrated in **Figure 1A**. An alternate-day gavage dosing protocol was applied (Yu et al., 2022). In the Con group, the mice were given PBS via gavage for a duration of 4 weeks as a control treatment. The Hp group were alternately gavaged 500 μL of *H. pylori* (2 × 10^9^ CFU/ml) suspension and 0.01 mmoL/L PBS solution. The Hp + CB group were alternately administered 500 μL of *H. pylori* (2 × 10^9^ CFU/ml) suspension and a volume of CB containing 3.3 g ethanol per kg body weight. This dosage is equivalent to a 20-g mouse ingesting 0.066 g of pure ethanol, which is approximately 0.084 mL (given the density of ethanol as 0.789 g/mL at room temperature of 20 °C). This volume corresponds to about 0.158 mL of CB (53% vol). The Hp + NACs group were alternately administered 500 μL of *H. pylori* (2 × 10^9^ CFU/ml) suspension or an equivalent amount of NACs. The experimental protocol stipulated the euthanizing of the mice after a 12-hour period of fasting at the culmination of the experiment. The gastric tissue was subsequently collected for qPCR and HE staining experiments. The cecal contents were collected for the purpose of gut microbiota analysis.

### 2.7 Histology

Immediately following collection, stomach tissue samples were promptly fixed in 4% formalin and then embedded in paraffin for later histological examination. Once the deparaffinization process was completed, sections measuring 4 µm in thickness underwent staining with hematoxylin and eosin (H&E). The images were captured by a Nikon DS-U3 camera.

### 2.8 DNA extraction and pyrosequencing

The DNA extracted from cecal contents was obtained using the TIANamp Micro DNA Kit (Tiangen, Beijing, China), adhering to the manufacturer’s protocols. After diluting the metagenomic DNA to a concentration of 1 ng/μL, amplification of the V3-V4 hypervariable region of the 16S rRNA gene was performed utilizing the primer pairs 338F (5’-ACTCCTACGGGAGGCAGCAG-3’) and 806R (5’-GGACTACHVGGGTWTCTAAT-3’). Sequencing of the PCR products was conducted on an Illumina MiSeq platform (PE250) at Shanghai Oebiotech Co., Ltd., China. After quality control, effective reads were organized into OTUs at a 97% sequence similarity threshold. The α-diversity was evaluated through indices such as Observed species, Shannon, Chao1, and Ace. For β-diversity assessment, PCoA was employed based on Bray-Curtis dissimilarity matrices. Differential bacterial analysis was conducted using linear discriminant analysis effect size (LEfSe) with a Linear Discriminant Analysis (LDA) score threshold of > 3.0. The functional prediction of the gut microbiota was accomplished utilizing PICRUSt2 software based on the sequencing dataset of the 16S rRNA gene (Douglas et al., 2020). The functional profiles at the third hierarchy level on KEGG pathways were examined utilizing STAMP v2.1.3 (Parks et al., 2014).

### 2.9 Non-targeted metabolomics

The qualitative analysis of NACs was conducted utilizing a Vanquish UHPLC System in conjunction with Orbitrap Exploris 120 mass spectrometry (Thermo Fisher Scientific, USA). A Waters ACQUITY HSS T3 column (100 × 2.1 mm id, 1.8 µm particle size) was used for chromatographic separation (Milford, MA, USA). A 2 μL injection volume was employed. The flow rate was held constant at 0.3 mL/min, with a column temperature maintained at 40 °C. In the case of the LC-ESI (+)-MS analysis, acetonitrile containing 0.2% formic acid (v/v) and water containing 0.1% formic acid (v/v) consisted the mobile phases. In contrast, the mobile phases for the LC-ESI (-)-MS analysis were mix of acetonitrile and ammonium formate at a concentration of 5 mM. The specified parameters for detection included sheath gas pressure of 40 arb, auxiliary gas flow of 10 arb, spray voltages set at 3.50 kV for ESI(+) and -2.50 kV for ESI(-). The capillary temperature was held at 325 °C, with an MS1 range extending from m/z 100 to 1000. The resolving power for MS1 was configured to 70,000 FWHM, while MS/MS had a resolving power of 17,500 FWHM. Additionally, the normalized collision energy was set to 30%, and the dynamic exclusion time was programmed to operate automatically. The mzXML format was achieved from the original data sets using MSConvert, part of the ProteoWizard software package. Following this conversion, the data underwent processing with R XCMS (v3.12.0) to perform tasks like feature extraction, adjustment of retention time, and data alignment. For the identification of metabolites, the accurate mass and MS/MS spectra were compared against several databases. These databases included HMDB, MassBank, LipidMaps, mzCloud, KEGG, and proprietary metabolite database established by Panomix Biomedical Tech Co., Ltd. based in Suzhou, China.

### 2.10 Statistical analysis

The concentrations of the analytes shown in the figures are represented as mean ± SD. To assess the differences between the various groups, a one-way analysis of variance (ANOVA) was conducted, subsequently followed by a Student’s *t*-test (SPSS 24.0, SPSS Inc.).

## 3 Results

### 3.1 Evaluation of the inhibitory effect of CB and NACs on *H*. *pylori*

**Figure 2:**
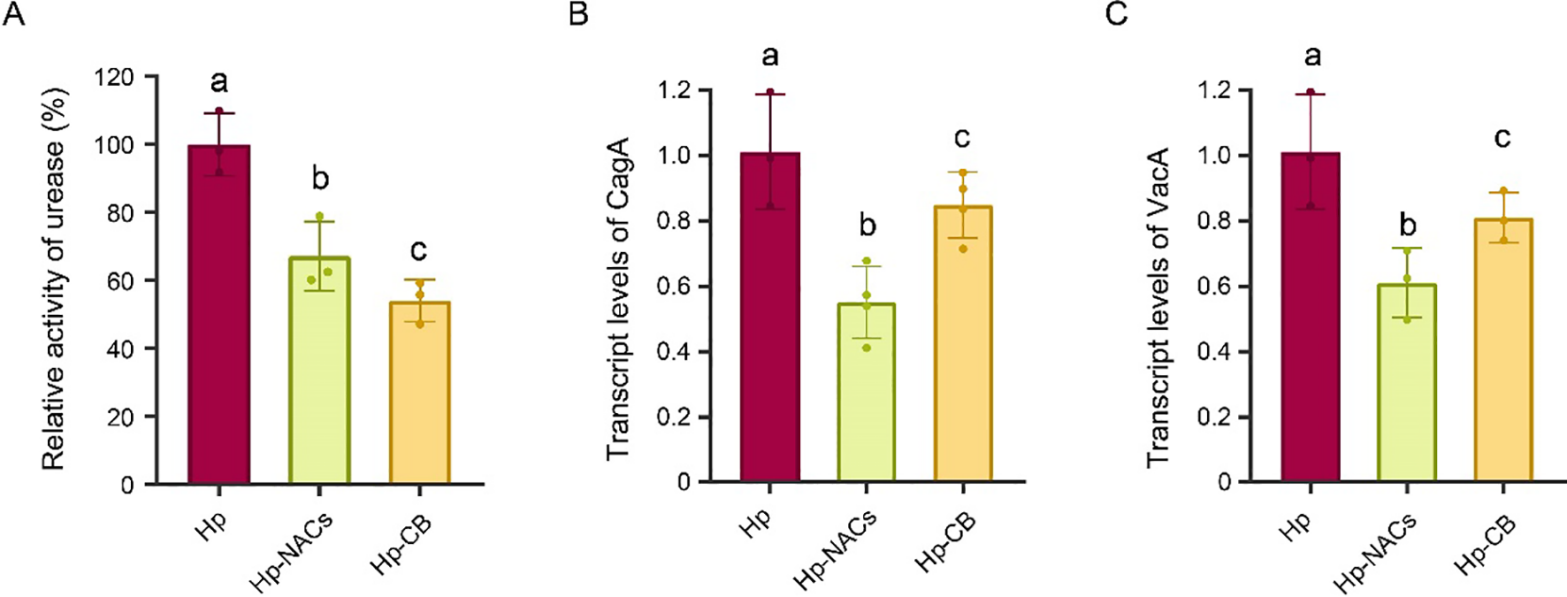
Effect of CB and NACs on the urease activity and transcription of virulence genes of *H. pylori*. **(A)** The urease activity. **(B)** The transcript levels of CagA. **(C)** The transcript levels of VacA. Hp, *H. pylori* group; Hp-NACs, non-alcoholic compounds treatment group. Hp-CB, Chinese Baijiu treatment group. Different lowercase letters above the bars indicate a statistically significant difference between groups (one-way ANOVA, P < 0.05); bars sharing the same lowercase letter indicate no significant difference between the corresponding groups.

To evaluate the inhibitory effect of CB and NACs on *H. pylori*, first, the MIC test was applied to determine their bacteriostatic activity. The results indicate that both CB and NACs exhibit bacteriostatic effect against *H. pylori* with the MIC of 156 mg/L (**Supplementary Table 2**). *H. pylori* can secrete urease and convert urea into ammonia, thereby neutralizing the acidic environment and ensuring its survival within the stomach milieu (Ansari and Yamaoka, 2017). CagA and VacA are capable of modulating cytokine and inflammatory signaling, as well as the innate immune cell function (Wizenty and Sigal, 2025). Thus, we next detected the urease activity and the transcript levels of CagA and VacA of *H. pylori* treated by effect of CB or NACs at 1/2 MIC (78 mg/L). The results indicate that both CB and NACs showed substantial urease-inhibited effect (**Figure 2A)** and reduced the transcript levels of CagA (**Figure 2B**) and VacA (**Figure 2C**). Furthermore, the NACs treatment had a significantly lower level of CagA and VacA expression compared to the CB treatment. These results indicated that both CB and NACs directly inhibit *H. pylori*.

### 3.2 Effect of CB and NACs on *H*. *pylori*-induced inflammation in GES-1 cells

**Figure 3:**
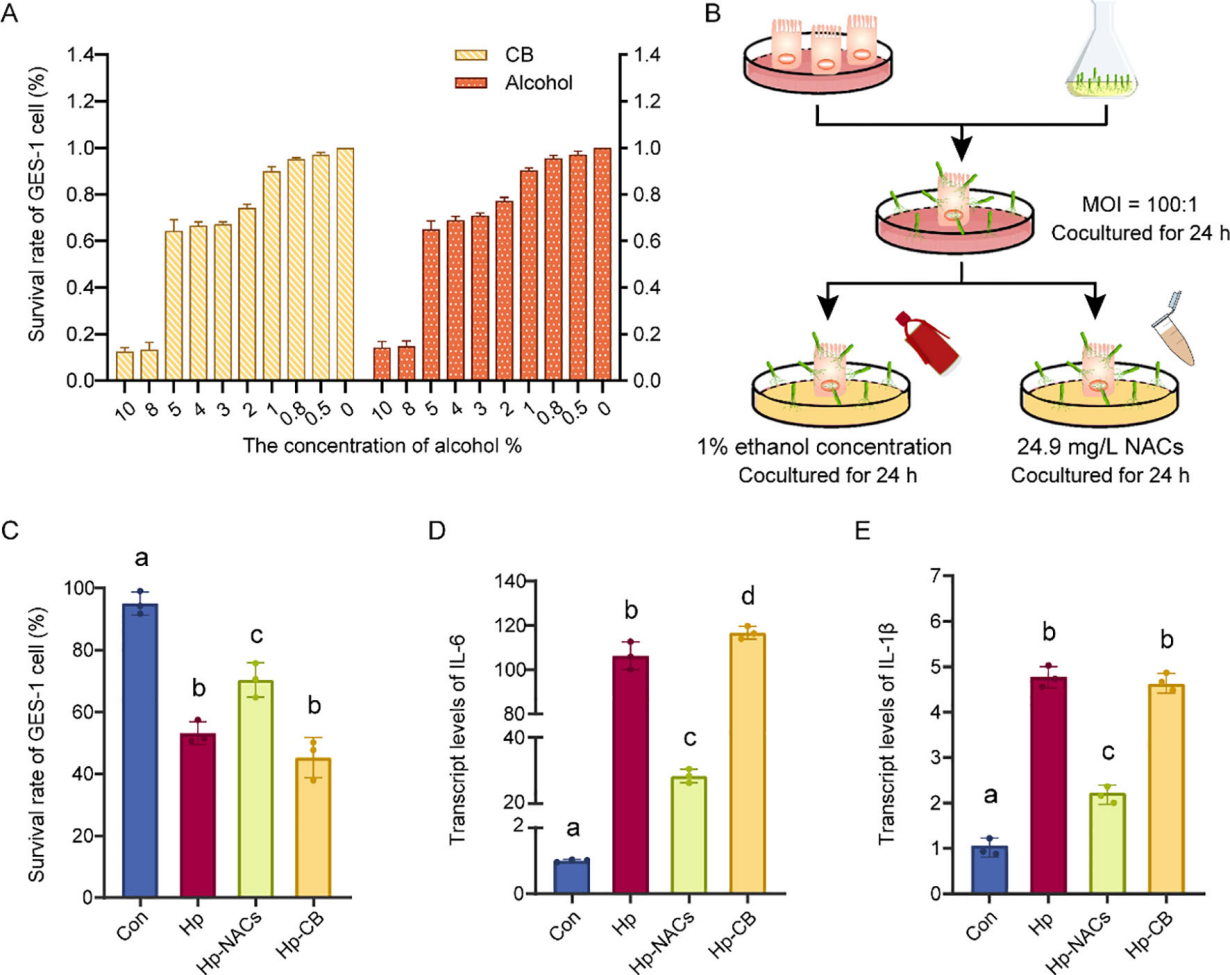
Effect of CB and NACs on *H*. *pylori*-induced inflammation in GES-1 cells. **(A)** survival rate of GES-1 cells. **(B)** Schematic diagram of cell experiments. **(C)** The survival rate of *H. pylori*-infected GES-1 cells treated with CB or NACs. **(D)** The transcript levels of IL-6. **(E)** The transcript levels of IL-1β. Different lowercase letters above the bars indicate a statistically significant difference between groups (one-way ANOVA, P < 0.05); bars sharing the same lowercase letter indicate no significant difference between the corresponding groups.

To establish a non-cytotoxic concentration of CB for subsequent cell infection experiments, and to distinguish the cytotoxic contribution of ethanol from the CB matrix, the CCK-8 assay was employed. The findings indicated that both CB and ethanol treatments over a period of 24 hours significantly reduced cell viability in a concentration-dependent manner (0, 0.5, 0.8, 1, 2, 3, 4, 5, 8, and 10% vol). The mean cell viability was 92.3 ± 3.1% at an alcohol concentration of 1%. When the alcohol concentration exceeds 1%, the cell viability is less than 90% (**Figure 3A**). Accordingly, CB of 1% alcohol concentration was selected as the optimal level for exploring its protective effects. GES-1 cells were co-cultured with *H. pylori* at an MOI of 100:1 for 24 h, after which they were treated with 1% ethanol concentration of CB or equivalent dose of NACs (24.9 mg/L, matching the NACs content in CB at 1% ethanol concentration) for a further 24 h (**Figure 3B**). As is shown in **Figures 3C–E**, although all the Hp treatment group reduced the cell viability (**Figure 3C**) and increased the mRNA expression of IL-6 **(Figure 3D**) and IL-1β (**Figure 3E**), Hp-NACs treatment group had higher cell viability and lower mRNA expression of IL-6 and IL-1β than that of Hp and Hp-CB group. These results indicated that NACs treatment inhibited the cytotoxicity of *H. pylori* against GES-1. To further confirms whether the observed anti-inflammatory effect of NACs was specific to *H. pylori* infection-induced inflammation, we evaluated the effect of CB (1% ethanol concentration) and NACs (24.9 mg/L) alone on GES-1 cell viability and transcript levels of pro-inflammatory cytokines (**Supplementary Figure 1**). As expected, CB and NACs alone had no significant effect on GES-1 cell viability, and did not induce the expression of IL-6 and IL-1β in normal GES-1 cells. These results confirmed that the anti-inflammatory effect of NACs was specific to *H. pylori* infection-induced inflammatory response, rather than a non-specific effect on normal gastric epithelial cells.

### 3.3 *In vivo* anti-*H. pylori* activity of CB and NACs

Next, we established an *H. pylori*-infected mouse model to determine whether NACs or CB could alleviate *H. pylori*-induced inflammation (**Figure 1A**).

Histological analysis using H&E staining revealed that the Hp and Hp-CB mice exhibited notable alterations in the structure of the gastric mucosa, along with considerable infiltration of immune cells. In comparison, the gastric tissue architecture remained more intact in the Hp-NACs group (**Figure 1B**). Furthermore, NACs supplementation notably reduced the increase in mRNA levels of CagA (**Figure 1C**) and VacA (**Figure 1D**) induced by *H. pylori* infection. In alignment with the findings from the *in vitro* study, NACs supplementation also significantly reduced the inflammatory response triggered by *H. pylori* infection, as revealed by the detection of transcript levels of IL-6 (**Figure 1E**) and IL-18 (**Figure 1F**). An increasing amount of evidence suggests that *H. pylori* infection can result in the impairment of the gastric barrier (Choi et al., 2022). Thus, the gastric barrier-related protein was also evaluated. Our results indicated that the expression of tight junction protein Occludin was downregulated by *H. pylori* infection, whereas NACs effectively increased its expression (**Figure 1G**).

### 3.4 *H. pylori* infection influences the gut microbial community structure

**Figure 4:**
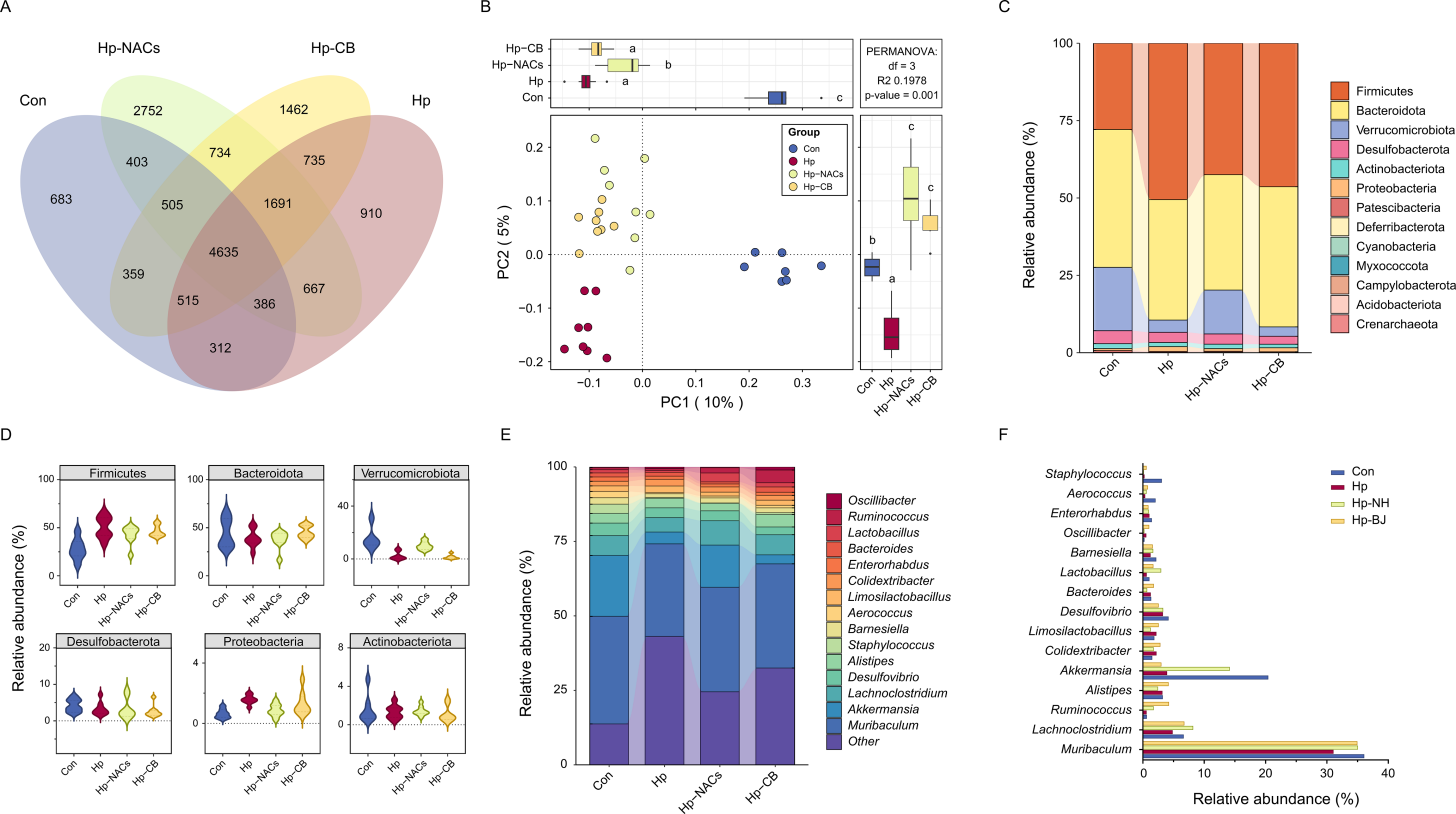
Effect of *H. pylori* infection on the composition of gut microbiota. **(A)** OUT numbers-based venn diagram. **(B)** Bray-Curtis distance-based PCoA. **(C)** A histogram depicting the community barplot analysis at the phylum level. **(D)** Phylums with an abundance greater than 1%. **(E)** A histogram illustrating the community barplot analysis at the genus level. **(F)** Barplot showing the top 14 genera. Different lowercase letters above the bars indicate a statistically significant difference between groups (one-way ANOVA, P < 0.05); bars sharing the same lowercase letter indicate no significant difference between the corresponding groups.

Accumulating evidence indicates that gut microbiota is crucial in modulating intestinal immunity (Honda and Littman, 2016). Recent studies have underscored the continual interaction between *H. pylori* and the gut microbiota. *H. pylori* not only influence the structure of the gastric microbial community but also exerts systemic influences that modify the composition of distal gut microbiota (Chen et al., 2021). In light of this, we explored whether the protective effects of NACs against inflammation induced by *H. pylori* infection are linked to changes in the gut microbiota. A total of 1,448,227 cleaned sequence reads were obtained from the 31 cecal samples by pyrosequencing. Rarefaction curves plateaued as the number of reads increased indicated that the majority of microbial diversity had been captured (**Supplementary Figure 2A**). The OTU number-based venn diagram revealed that a total of 16,749 OTUs obtained. Amongst these OTUs, 4635 were shared by both groups, indicating a robust core microbial community (**Figure 4A**).

Compared with the control mice, all the experimental mice displayed significant increased observed species (**Supplementary Figure 2B**) and microbial diversity (Shannon index) (**Supplementary Figure 2C**). Furthermore, Hp and Hp-CB mice also displayed substantially increased microbial richness (Ace index) (**Supplementary Figure 2D**) and number of OTUs (Chao1 index) (**Supplementary Figure 2E**), while Hp-NACs gavage mitigated the increase. Bray-Curtis distance-based PCoA showed a significant separation in microbiota structure among the four groups (**Figure 4B**). Compared to the control mice at the phylum level, a higher proportion of Firmicutes and Proteobacteria was observed in all experimental mice (**Figures 4C, D**), along with an elevated ratio of Firmicutes to Bacteroidetes (**Supplementary Figure 2F**). Moreover, although all the experimental mice decreased proportion of Verrucomicrobia (**Figures 4C, D**), its proportion in the Hp-NACs group was significantly higher than that of Hp and Hp-CB group. At the genus level, colonization of *H. pylori* reduced relative abundance of *Lachnoclostridium*, *Akkermansia*, *Lactobacillus*, *Aerococcus*, and *Staphylococcus*. Whereas, CB and NACs supplementation increased the relative abundance of *Lactobacillus*, and protect the reduction of *Lachnoclostridium*. NACs supplementation also prevented the reduction of *Akkermansia* (**Figures 4E, F**).

### 3.5 Differentially abundant gut microbiota and functional prediction analysis

**Figure 5:**
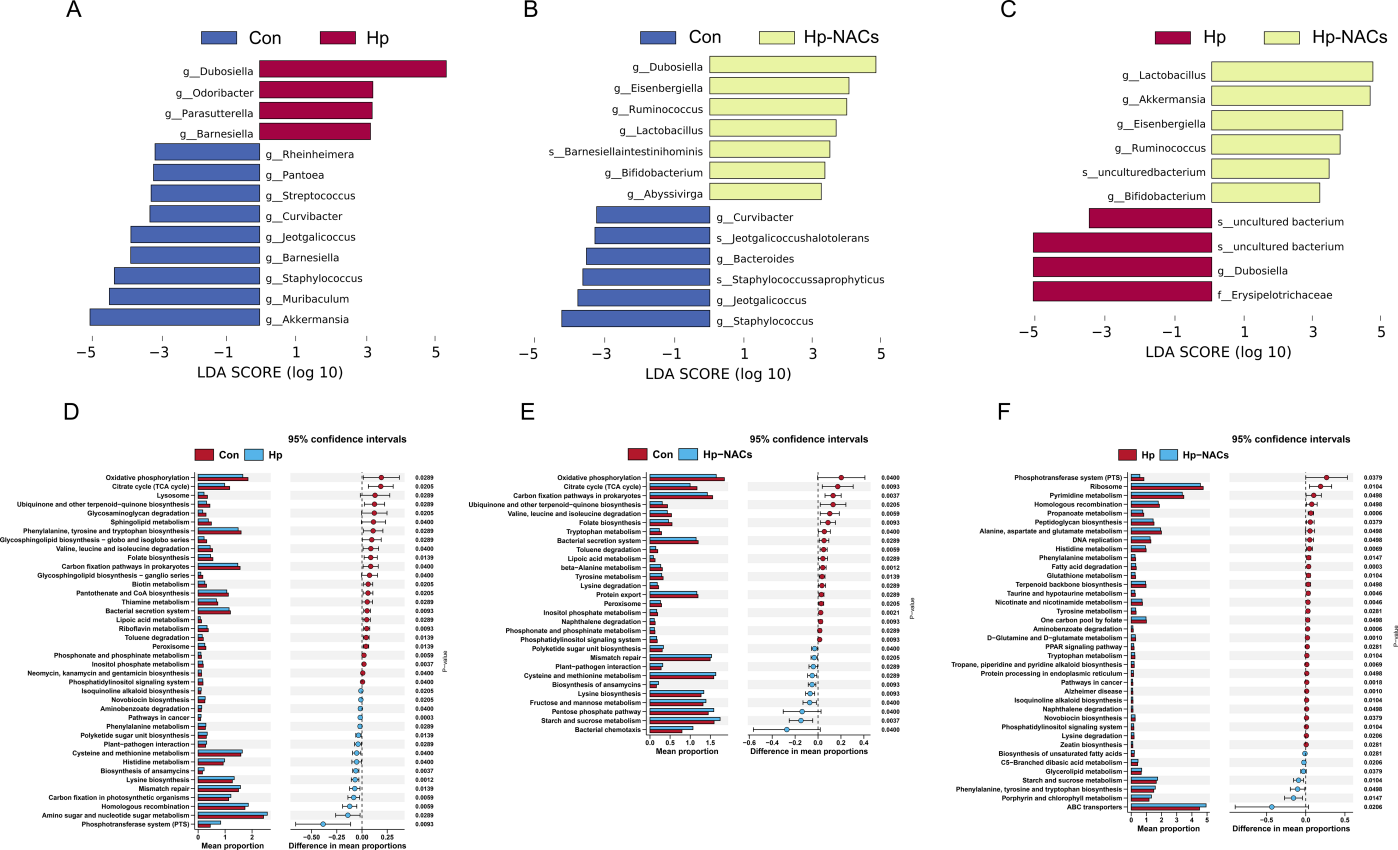
Differentially abundant gut microbiota and potential pathways. **(A)** Comparison of bacterial abundance between Con and Hp group. **(B)** Comparison of bacterial abundance between Con and Hp-NACs group. **(C)** Comparison of bacterial abundance between Hp and Hp-NACs group. Only taxa with LDA score greater than 3 are shown. **(D)** Pathways with differential abundance between Con and Hp group. **(E)** Pathways with differential abundance between Con and Hp-NACs group. **(F)** Pathways with differential abundance between Hp and Hp-NACs group. Differential functional predictions were determined through STAMP analysis, with 95% confidence intervals (Wilcox test, *P* < 0.05 were shown).

To further screen out the key differential bacterial genera, we performed pairwise LEfSe analysis among the Con, Hp and Hp-NACs group. Compared to the control mice, Hp colonization was enriched in the genera *Dubosiella*, *Odoribacter*, *Parasutterella*, and *Barnesiella* (**Figure 5A**). Hp-NACs group was enriched in the genera *Dubosiella*, *Eisenbergiella*, *Ruminococcus*, *Lactobacillus*, *Bifidobacterium*, and *Abyssivirga* (**Figure 5B**). Interestingly, we also observed that the Hp-NACs treatment was enriched in the genera *Lactobacillus*, *Akkermansia*, *Eisenbergiella*, *Ruminococcus*, and *Bifidobacterium* when compared to the Hp mice (**Figure 5C**).

To further understand the potential impact of the alteration of gut microbiota to function, we used PICRUSt2 to predict microbial functions of different bacterial communities. A comparison of Hp mice and control mice revealed significant enrichments in pathways related to cancer, biosynthesis of ansamycins, lysine biosynthesis, and the phosphotransferase system (PTS). Concurrently, there were reduced levels of pathways involved in riboflavin metabolism, phosphonate and phosphinate metabolism, and inositol phosphate metabolism (*P* < 0.01) (**Figure 5D**). In Con group and Hp-NACs group, the major disparate pathways included biosynthesis of ansamycins, lysine biosynthesis, starch and sucrose metabolism, inositol phosphate metabolism, naphthalene degradation, beta-alanine metabolism, toluene degradation, folate biosynthesis, citrate cycle (TCA cycle) and carbon fixation pathways in prokaryotes (*P* < 0.01) (**Figure 5E**). It is noteworthy that a discernible discrepancy was evident between the Hp and Hp-NACs groups. In comparison with the Hp group, the Hp-NACs group exhibited a substantial decrease in the levels of Alzheimer disease, pathways in cancer, D-glutamine and D-glutamate metabolism, tropane, piperidine and pyridine alkaloid biosynthesis, aminobenzoate degradation, taurine and hypotaurine metabolism, nicotinate and nicotinamide metabolism, fatty acid degradation, histidine metabolism and propanoate metabolism (*P* < 0.01) (**Figure 5F**).

### 3.6 Non-targeted metabolomics approach-based compositional analysis of the NACs

**Figure 6:**
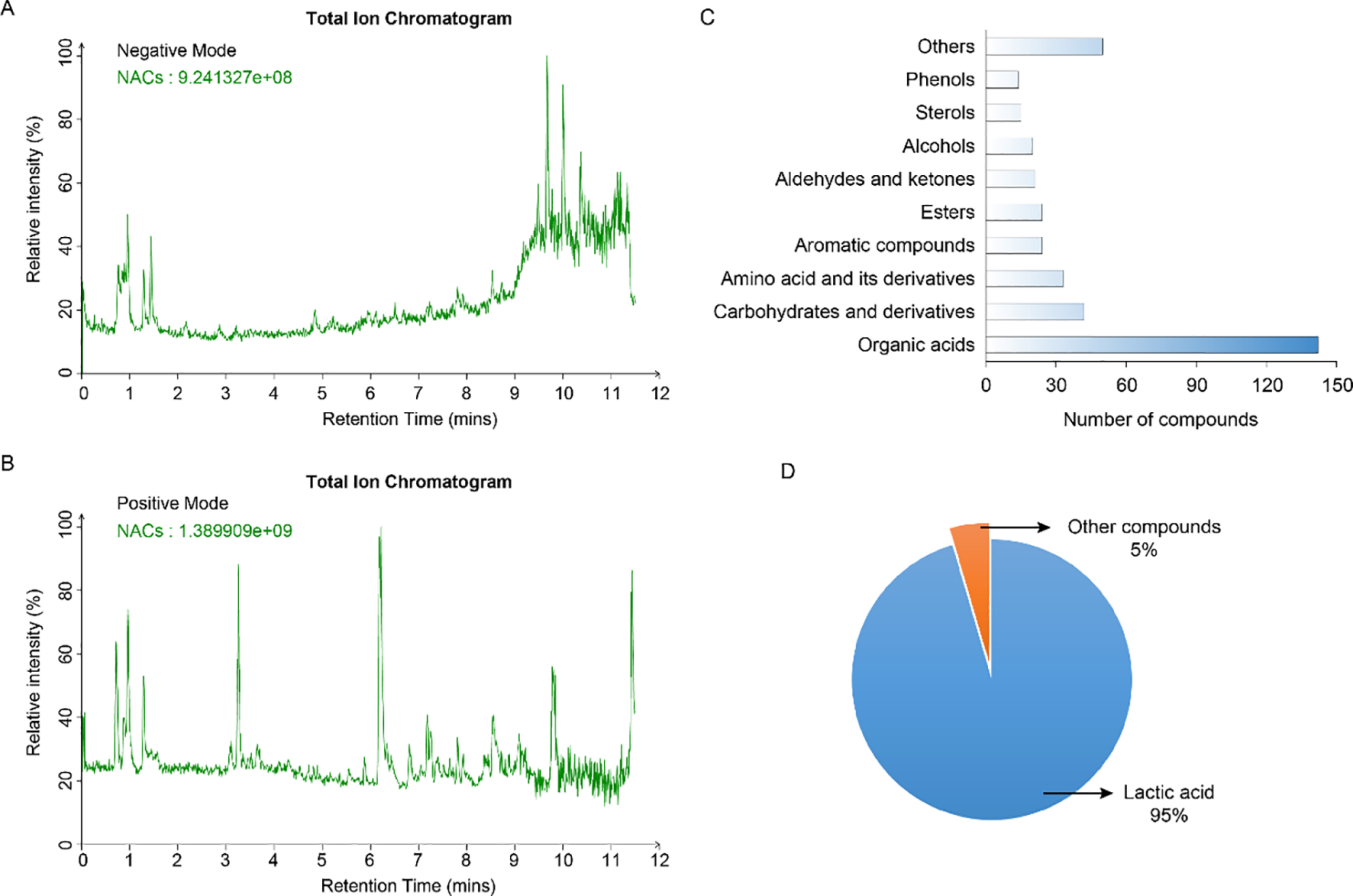
Compositional analysis of the NACs **(A)** The TICs in negative ion mode. **(B)** The TICs in positive ion mode. **(C)** The number and class of compounds detected in NACs. **(D)** The proportion of lactic acid in the total NACs content.

To understand antibacterial mechanism of NACs, a UHPLC-MS-based non-targeted metabolomics approach was applied to analyze the chemical composition of the NACs. The HPLC-MS total ion chromatograms (TICs) obtained for the NACs, from both ionization conditions (ESI– and ESI+), are shown in **Figures 6 A, B**. By comparing the accurate mass and MS/MS spectra against a variety of databases, a total of 384 compounds were identified. These compounds included 142 organic acids, 41 carbohydrates and derivatives, 33 amino acid and derivatives, 24 aromatic compounds, 24 esters, 21 aldehydes and ketones, 20 alcohols, 15 sterols, 14 phenols (**Figure 6C**; **Supplementary Table 3**). A KEGG analysis revealed that these compounds are implicated in a variety of physiological functions, including amino acid and energy metabolism, signal transduction, structural support, biosynthesis of secondary metabolites, and more (**Supplementary Table 3**). Notably, a multitude of substances were identified in the CB for the first time, such as kojic acid, norlinolenic acid, nicotinic acid, pimelic acid, 4-hydroxycinnamic acid, gitogenin, catechol, etc. It has been documented that lactic acid is the predominant non-volatile compounds in CB (Luo, 2023). Thus, we further quantitatively analyzed its content. As anticipated, the lactic acid concentration in CB employed in this study was found to be as high as 1.26 g/L, which constituted 95% of the total NACs (**Figure 6D**).

### 3.7 The *in vitro* anti-*H*. *pylori* activity of lactic acid

**Figure 7:**
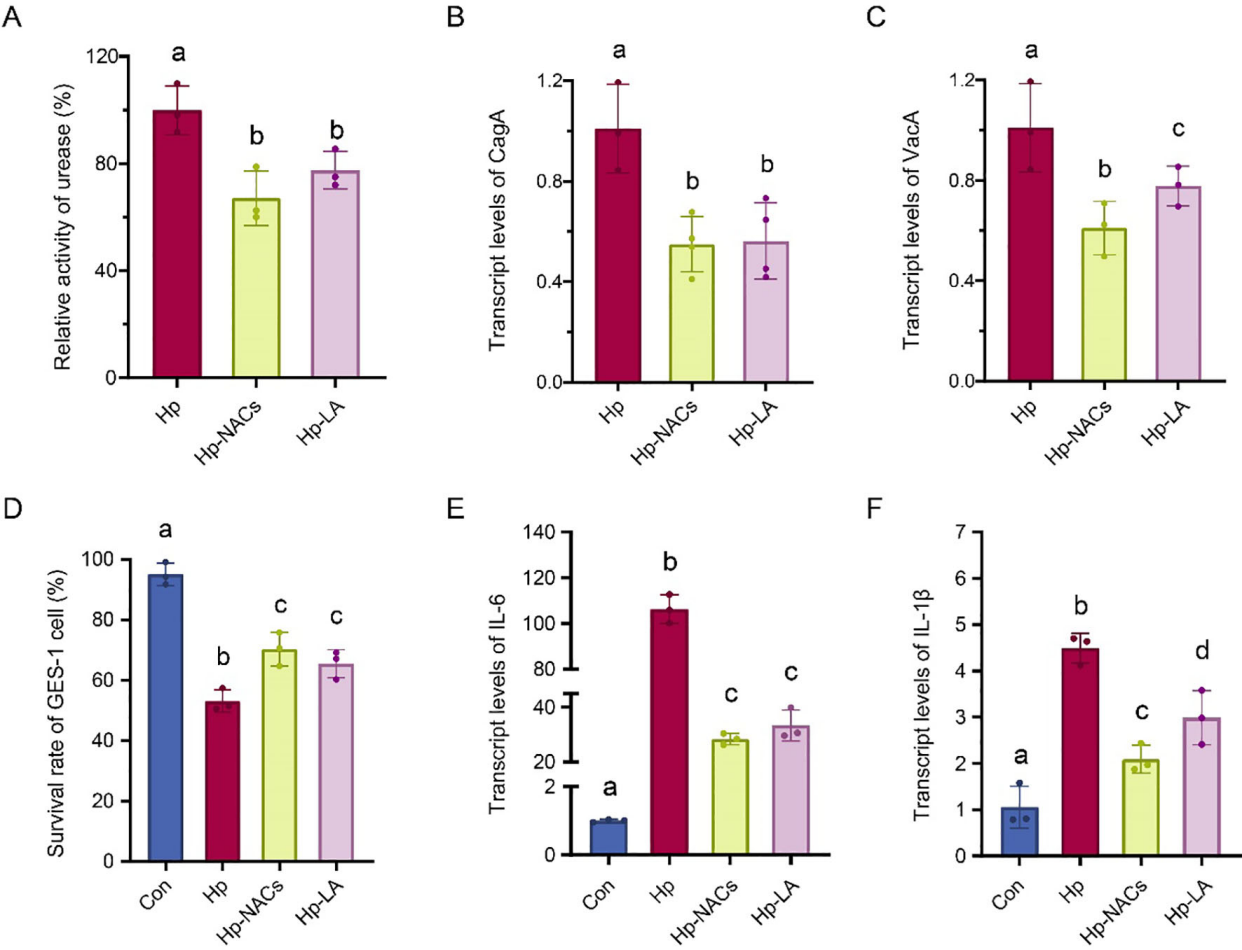
The *in vitro* activity of lactic acid against *H*. *pylori*. **(A)** The urease activity. **(B)** Transcript levels of CagA. **(C)** Transcript levels of VacA. **(D)** GES-1 cell survival rate. **(E)** Transcript levels of IL-6. **(F)** Transcript levels of IL-1β. Different lowercase letters above the bars indicate a statistically significant difference between groups (one-way ANOVA, P < 0.05); bars sharing the same lowercase letter indicate no significant difference between the corresponding groups.

In order to ascertain the role of lactic acid in the observed antibacterial effects and to determine whether other compounds in NACs exhibit synergistic effects, we treated *H. pylori* with lactic acid alone or NACs. Although both lactic acid and NACs exhibited clear anti-*H. pylori* effect, lactic acid (312.5 mg/L) demonstrated a higher MIC in comparison to NACs treatment (156.25 mg/L) (**Supplementary Table 4**). Furthermore, lactic acid exhibited a substantial urease-inhibiting effect (**Figure 7A**) and reduced the transcript levels of CagA (**Figure 7B**) and VacA (**Figure 7C**). However, its inhibitory effect was less potent than that of NACs. Furthermore, the *in vitro* protective effects of lactic acid on *H. pylori* infection-induced inflammation were investigated. As expected, lactic acid treatment resulted in higher cell viability (**Figure 7D**) and reduced mRNA expression of IL-6 (**Figure 7E**) and IL-1β (**Figure 7F**) in comparison to the Hp group. Nevertheless, NACs exhibited more superior inhibitory effects. Collectively, lactic acid contributed to the main anti-*H. pylori* potential of NACs and other compounds in NACs exhibit synergistic effects.

## 4 Discussion

Researches on the association between *H. pylori* infection and alcohol consumption have attracted significant attention and yielded contradictory results. Nonetheless, it is widely accepted that moderate alcohol intake has a positive impact on the elimination of *H. pylori* (Murray et al., 2002; Kuepper-Nybelen et al., 2005). Conversely, excessive alcohol consumption aggravates the *H. pylori* infect by compromising the integrity of the gastric mucosa and consequently impairing the gastric mucosal defense barrier (Zhang et al., 2010; Du et al., 2023). In addition, excessive alcohol consumption may lead to gut microbiota dysbiosis and further damage mucosal barrier (Stärkel et al., 2018). Given the important role of gut bacteria in immune regulation (Honda and Littman, 2016), excessive alcohol consumption may also exacerbate a *H. pylori* infection by inducing gut dysbiosis. Therefore, in this study, the ethanol dosage delivered via CB for the mouse model was set at 3.3 g ethanol/kg body weight. This dosage was translated from human moderate drinking. The Food and Drug Administration (FDA) has been recommended that the dose in mice is assumed to be 12.3 times higher than the dose required in adult humans to yield similar biological effects (Reagan-Shaw et al., 2008). Thus, the mouse dosage of CB (containing 3.3 g ethanol/kg body weight) is equivalent to approximately 0.27 g/kg for humans. This is analogous to a 75-kilogram adult consuming 20.25 grams of alcohol, which falls within the recommended moderate drinking (using NIH/CDC definitions: 14 g ethanol per standard drink) in the Dietary Guidelines (Dufour, 1999).

Gut microbiota is one of the pivotal factors closely correlated with host disease and health (Fan and Pedersen, 2021). *H. pylori* infection can alter the local microenvironment and induce chronic inflammatory response (Chen et al., 2024). However, the impact of *H. pylori* infection on distant gut microbiota, which is important in maintaining host immune homeostasis, remains to be fully elucidated. Here, our results demonstrated that *H. pylori* infection changed cecal gut microbiota structure. The NACs in CB can ameliorate *H. pylori* infection induced inflammation, partially restore the alteration of gut microbiota composition and promote the growth of beneficial gut commensal bacteria.

An intriguing finding was the effect of *H. pylori* infection and NACs intervention on α-diversity of gut microbiota. Contrary to the typical pattern in many gastrointestinal diseases where diversity decreases (Wang et al., 2023), *H. pylori* colonization led to an increase in all indices of α-diversity (**Supplementary Figures 1B–E**). This may represent a state of dysbiotic instability or community disruption. Notably, NACs intervention mitigated the infection-induced increase in richness (Chao1, Ace indices) while maintaining a high Shannon diversity index, suggesting a role in restoring microbial community stability rather than merely reducing diversity. This aligns with the emerging understanding that a healthy gut ecosystem is defined by both diversity and resilience (Fassarella et al., 2021).

Previous studies suggested that the non-alcoholic compounds in Baijiu can regulate gastrointestinal microbiota homeostasis by protecting reduction of the beneficial commensal bacteria while suppressing harmful bacteria (Fang et al., 2019b, 2022b, 2025). In present study, we observed that supplementation of NACs enriched in the genera *Lactobacillus*, *Bifidobacterium*, *Dubosiella*, *Eisenbergiella*, *Ruminococcus* and *Abyssivirga*. *Lactobacillus* and *Bifidobacterium* a are well recognized for their probiotic applications, which are utilized in the treatment of gastrointestinal inflammation-related diseases, including gastroenteritis, irritable bowel syndrome, inflammatory bowel diseases, and allergies (Cizeikiene and Jagelaviciute, 2021). Additionally, the administration of probiotics has been demonstrated to enhance the eradication and reduce the occurrence of adverse events such as diarrhea produced by standard therapy (Oh et al., 2016). *Dubosiella*, *Eisenbergiella* and *Ruminococcus* are both robust short chain fatty acid (SCFA)-producing commensal bacteria and linked to a reduced risk of inflammation-associated diseases (Breuninger et al., 2021; Xu et al., 2024; Zhang et al., 2024b). For instance, *Dubosiella* has been demonstrated to alleviate colitis induced by dextran sulfate sodium in mouse models by rebalancing Treg/Th17 responses and improving mucosal barrier function (Zhang et al., 2024b). *Eisenbergiella* is clinically linked to a reduced risk of periodontitis (Xu et al., 2024).

In addition to the genera previously mentioned, we also observed significant higher abundance of *Akkermansia* in the Hp-NACs mice compared to the Hp mice. Despite the underlying mechanism is largely limited, researches have shown that *H. pylori* infection leads to a reduction in *Akkermansia*, as evidenced by both epidemiological and animal model studies (White et al., 2021; Tandoro et al., 2025). Thus, our results indicated that the NACs can protect against the reduction in *Akkermansia* abundance induced by *H. pylori* infection. *Akkermansia* is the only genus with in phylum Verrucomicrobia. It has been estimated that there are at least four species within this genus (Ioannou et al., 2025). *A. muciniphila* is the earliest discovered and most extensively studied species. It was recognized as the paradigm for next-generation beneficial microorganisms (Cani et al., 2022). Recent efforts suggested that *A. muciniphila* may stimulate intestinal adaptive immune responses during homeostasis (Ansaldo et al., 2019) and the cell membrane phospholipid plays a vital role during this process (Bae et al., 2022). Drawing from the available evidence, we hypothesize that the inhibitory role of the NACs on *H. pylori* may be regulated by promoting the growth of *A. muciniphila*.

Otherwise, the microbial functions were predicted based on the microbiota abundance and KEGG database. Compared to the control group, the Hp group exhibited a greater abundance in the pathways associated with cancer. This finding aligns with the results from a previous clinical study conducted by Miao et al (Miao et al., 2020). The upregulation of pathway in cancer corresponds with the clinical pathogenicity associated with *H. pylori*, known to potentially lead to gastric cancer. Of our particular interest is the observation that the abundance of pathways in cancer was also lower in the Hp-NACs group than that of the Hp group. In addition to pathways in cancer, we observed a significant lower abundance of pathways associated with Alzheimer disease in the Hp-NACs group compared to the Hp group. In light of the epidemiologic and experimental evidence suggesting a potential influence of *H. pylori* infection on the progression of Alzheimer’s disease (Doulberis et al., 2018), these findings provides indirect support for the potential beneficial effect of NACs on *H. pylori* infection-related systemic disorders. Nonetheless, these results do not indicate that NACs have direct therapeutic effects on cancer or Alzheimer’s disease. Further in-depth studies are needed to verify the causal relationship. Following NACs treatment in mice infected with *H. pylori*, we also observed multiple alteration in pathways associated with alkaloid metabolism (tropane, piperidine and pyridine alkaloid biosynthesis, nicotinate and nicotinamide metabolism), amino acid metabolism (aminobenzoate degradation, histidine metabolism), lipid metabolism (fatty acid degradation, propanoate metabolism) and carbohydrate metabolism (D-glutamine and D-glutamate metabolism). These results suggested a pleiotropic regulatory effect of Baijiu components on gut microbiota function (Fang et al., 2022a, b, 2025). Collectively, the regulation of these pathways by NACs may be related to the control of *H. pylori* infection.

It has been proposed that the distinctive solid-state distillation process inherent to Baijiu production gives rise to the presence of a considerable number of NACs in conjunction with the volatiles (Fang et al., 2019a). However, the research interest concerning the compositional study of Baijiu was primarily focused on the volatile substances that contribute to flavor of Baijiu in recent decades (Xu et al., 2022). The NACs have historically been overlooked, despite the fact that many of these compounds are bioactive (Kang et al., 2023). In this study, we employed an untargeted metabolomics approach to analyze the NACs, with the aim of elucidating the molecules that contribute to the amelioration of *H. pylori* infection. As expected, we detected a series of microbial metabolites with substantial anti-inflammatory and antibacterial properties, such as andrographolide (Ala’a et al., 2009), azelaic acid (Ismail et al., 2024), 4-hydroxybenzoic acid (López-Herrador et al., 2025), tyrosol (Muriana et al., 2017), hydrocinnamic acid (Devi et al., 2010), gallic acid and ferulic acid (Borges et al., 2013), among others. Azelaic acid, 4-hydroxybenzoic acid and tyrosol are the bioactive metabolites of *Fusarium solani* (Ismail et al., 2024), a fungus that has been isolated from fermented grain of Baijiu production (Zhang et al., 2024a). Hydrocinnamic acid, gallic acid and ferulic acid can be produced by bacilli strains (Devi et al., 2010; Aguilar-Zárate et al., 2015; Duan et al., 2021), which was widely exist in Baijiu production. Beyond the identified bioactive metabolites mentioned above, the anti-*H. pylori* effect of NACs may also closely link to the physiological acidic environment of the stomach, which is the core niche for *H. pylori* survival and colonization. The urease-mediated hydrolysis of urea to produce ammonia, which forms a neutralized periplasmic microenvironment to protect *H. pylori* from the bactericidal effect of gastric acid, is well-established as one of the core survival mechanisms enabling *H. pylori* to colonize the harsh acidic gastric lumen. Our *in vitro* results confirmed that NACs significantly inhibit the urease activity of *H. pylori*, which may block the bacteria’s ability to neutralize gastric acid, deprive *H. pylori* of its acid defense. Therefore, the NACs may exert a synergistic bacteriostatic effect with physiological gastric acid *in vivo*.

**Figure 8:**
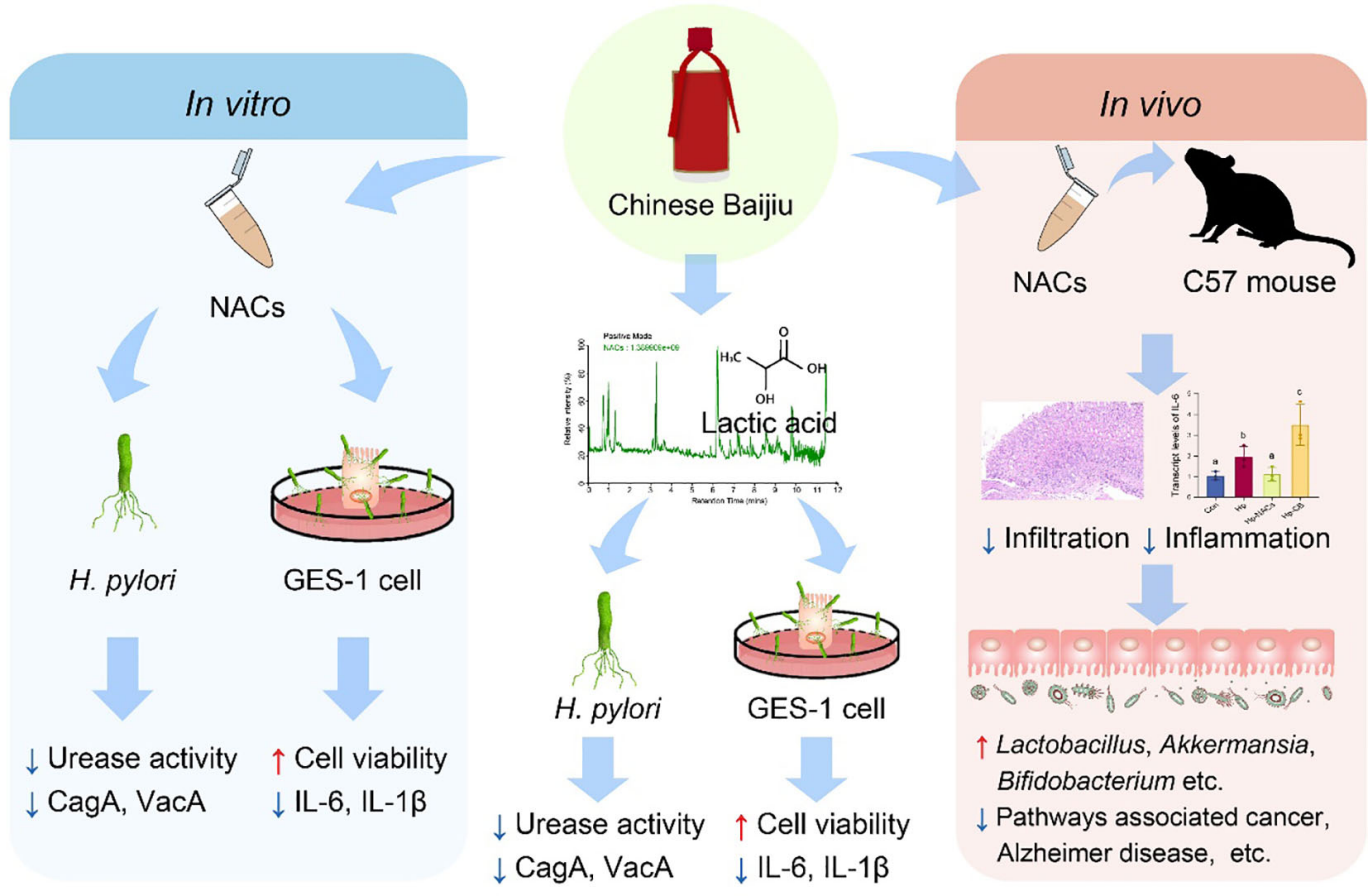
Suggested mechanism underlying the inhibitory action of NACs on *H. pylori*. Both *in vitro* and *in vivo* investigations demonstrated that NACs have notable anti-*H. pylori* properties. Mechanistically, NACs modulate the gut microbiota structure and potential function by enhancing the proliferation of beneficial gut commensal bacteria. Further compositional analysis coupled validation experiments indicated that lactic acid contributed to the anti-*H. pylori* potential of NACs.

As one of the most abundant NACs in CB, lactic acid is considered to be a major component responsible for the bacteriostatic functions of probiotics (Fijan, 2023). Thus, we quantified the concentration of lactic acid and further evaluated its inhibitory impact on *H. pylori in vitro*. As anticipated, a concentration of lactic acid equivalent to that found in baijiu (1.26 g/L) demonstrated a significant inhibitory effect on *H. pylori*, though the bacteriostatic effect was less potent than that of the NACs combination. In fact, previous studies have confirmed the anti-*H. pylori* potential of lactic acid both *in vitro* and *in vivo* (Aiba et al., 1998; Sgouras et al., 2004). In the mouse model conducted by Aiba et al., they showed that orally administration of 9 mg/day lactic acid can sufficiently inhibit the growth of *H. pylori*. The population of *H. pylori* decreased to one-tenth in the mice after 4-weeks treatment with lactic acid (Aiba et al., 1998). Sgouras et al. observed that more than 70% of the urease activity was inhibited within three hours when incubation of *H. pylori* with lactic acid at a concentration of 15 mM, which is equivalent to 1.35 g/L (Sgouras et al., 2004). Mechanistically, lactic acid acts as a permeabilizer of the cell membrane in Gram-negative bacteria and may synergize with other antimicrobial substances in NACs (Alakomi et al., 2000). In addition to lactic acid, other organic acids have recently been shown to inhibit *H. pylori* through a similar mechanism (Gao et al., 2025). Malic and tartaric acids, which were also detected in the NACs, exhibited notable anti-*H. pylori* properties. They functioned by preventing biofilm formation, increasing the permeability of the outer membrane, disrupting membrane integrity, reducing urease activity, and modifying membrane protein conformation (Gao et al., 2025). Therefore, while the precise mechanism by which lactic acid and other organic acids in NACs inhibits *H. pylori* remains elusive, both our findings and those of preceding research suggested that this effect is most likely attributable to its inhibitory effect on the bacterial urease system and the its capacity to disrupt the outer membrane of Gram-negative bacteria. While our study demonstrated the anti-*H. pylori* properties of NACs and proposed the potential mechanisms from the perspective of gut microbiota (**Figure 8**), it is important to recognize certain limitations. Firstly, we noted a marked increase in the levels of several beneficial commensal bacteria following the NACs intervention. Nevertheless, the question of whether this increase is attributable to lactic acid remains unresolved. It is necessary to design mouse model to further study the impact of lactic acid on the gut microbiota. Secondly, the unknown content of other active ingredients beyond lactic acid has led to insufficient understanding of the dose-response relationships for these components. Subsequent studies should perform accurate quantitative analysis on these components. Furthermore, although it was observed that NACs can inhibit urease activity and reduce the transcription levels of CagA and VacA at the minimum inhibitory concentration, the minimum bactericidal concentration (MBC) and time-kill curves for NACs remain unknown. Future studies could employ MBC and time-kill kinetic assays to more precisely characterize the temporal antibacterial dynamics of NACs and its key components against *H. pylori*. Finally, we proposed that the NACs prevent *H. pylori* infection-induced decrease of *Akkermansia*. However, it is still unclear whether *Akkermansia* supplementation can inhibit *H. pylori* infection. Furthermore, given the established role of *Akkermansia* in immune regulation, the possibility of a systemic mechanism with an immunological basis should not be overlooked. Further *in vivo* studies are necessary to explore the effects of *Akkermansia* treatment on *H. pylori* infection.

## 5 Conclusion

In conclusion, our study showed that NACs can ameliorate *H. pylori* infection-induced inflammation both *in vitro* and *in vivo*. In addition to directly inhibiting urease activity and transcription of virulence genes in *H. pylori* and its infection-induced increase of inflammation, NACs restored the gut dysbiosis induced by *H. pylori* infection through promoting the growth of beneficial gut commensal bacteria and reducing potential intestinal pathogens. Further compositional analysis coupled validation experiments indicated that lactic acid contributed to the main anti-*H. pylori* potential of NACs. Our results provide a theoretical basis for the adjunctive treatment of *H. pylori* infection by food ingredients derived from spontaneous fermentation. Furthermore, it serves to expand upon the existing corpus of knowledge pertaining to the effect of *H. pylori* infection on the gut microbiota, a field of research which is as yet limited in scope.

## Data Availability

The original contributions presented in the study are included in the article/**Supplementary Material**. Further inquiries can be directed to the corresponding authors.
