# Supplementary material for: Inhibitory effect of non-alcoholic compounds from spontaneously fermented beverage on Helicobacter pylori
Source: Front Cell Infect Microbiol. 2026 Apr 24;16:1742545. doi: 10.3389/fcimb.2026.1742545 (PMC13153108; doi:10.3389/fcimb.2026.1742545)
Supplement: Supplementary file 1 [file Table1.docx]

***Supplementary Information***

# Inhibitory effect of non-alcoholic compounds from spontaneously fermented beverage on *Helicobacter pylori*

Cheng Fang ^a,1^, Ziyi Lei ^a,1^, Yehui Han ^a^, Pinghua Tang ^b^, Guanghui Yu ^b^, Jinyuan Sun ^c^, Bowen Wang ^c*^, Yan Xu ^a*^

^a^ Laboratory of Brewing Microbiology and Applied Enzymology, Key Laboratory of Industrial Biotechnology of Ministry of Education, School of Biotechnology, Jiangnan University, 1800 Lihu Avenue, Wuxi, 214122, Jiangsu, China

^b^ Institute of Renhuai Jiangxiang Baijiu, Renhuai, 564500, Guizhou, China

^c^ Key Laboratory of Geriatric Nutrition and Health (Beijing Technology and Business University), Ministry of Education, School of Food and Health, School of Light Industry Science and Engineering, Beijing Technology and Business University, Beijing, 100048, China

**Supplementary Tables**

**Table S1** Sequences of the primers used for RT-PCR.

| Genes | Primers | Sequences |
| --- | --- | --- |
| *H. pylori* specific gene | Forward primer | 5’-TTTGTTAGAGAAGATAATGACGGTATCTAAC-3’ |
|  | Reverse primer | 5’-CATAGGATTTCACACCTGACTGACTATC-3’ |
| *CagA* | Forward primer | 5’-ATTCCCTAGGGCGTCTAAATAA-3’ |
|  | Reverse primer | 5’-GTCATAATGGCATAGAACCTGAA-3’ |
| *VacA* | Forward primer | 5’-TGGCGAGCAGTTATTATTCCCT-3’ |
|  | Reverse primer | 5’-TGGCGAGCAGTTATTATTCCCT-3’ |
| *IL-1β* | Forward primer | 5’-AGAGCCCATCCTCTGTGACTCA-3’ |
|  | Reverse primer | 5’-TGCTTGGGATCCACACTCTCCA-3’ |
| *IL-6* | Forward primer | 5’-TTCGGTCCAGTTGCCTTCT-3’ |
|  | Reverse primer | 5’-TGAATTCTCAGCCCTCTTCAA-3’ |
| *GADPH* | Forward primer | 5’-TCTCCTGCGACTTCAACA-3’ |
|  | Reverse primer | 5’-TGTAGCCGTATTCATTGTCA-3’ |

**Table S2** Determination of minimum inhibitory concentration

| Concentration (mg/L) | Con | NACs | CB |
| --- | --- | --- | --- |
| 10000 | ○ | × | × |
| 5000 | ○ | × | × |
| 2500 | ○ | × | × |
| 1250 | ○ | × | × |
| 625 | ○ | × | × |
| 312.50 | ○ | × | × |
| 156.25 | ○ | × | × |
| 78.13 | ○ | ○ | ○ |
| 39.07 | ○ | ○ | ○ |
| 19.54 | ○ | ○ | ○ |

The symbol "○" is used to denote turbidity that is visible to the naked eye, whereas "×" indicates the absence of such turbidity.

**Table S3** Compounds detected in NACs

| **Compounds** | **Formula** | **CAS** | **KEGG** | **m/z** | **RT (s)** | **Precursor type** |
| --- | --- | --- | --- | --- | --- | --- |
| **Organic acids** | | | | | | |
| L-Glutamic acid | C5H9NO4 | 6893-26-1 | C00025 | 130.0494 | 256.8 | [M+H-H2O]+ |
| trans-trans-Muconic acid | C6H6O4 | 3588-17-8 | C02480 | 143.0383 | 162.6 | [M+H]+ |
| β-Glycerophosphoric acid | C3H9O6P | 17181-54-3 | C02979 | 171.0111 | 566.4 | [M-H]- |
| 3-(3-Hydroxyphenyl)propanoic acid | C9H10O3 | 621-54-5 | C11457 | 149.0595 | 345.4 | [M+H-H2O]+ |
| Isoferulic acid | C10H10O4 | 537-73-5 | C10470 | 177.0549 | 400 | [M+H-H2O]+ |
| Delta-12-Prostaglandin J2 | C20H30O4 | 87893-54-7 | C05958 | 334.2227 | 420.9 | [M]+ |
| Prostaglandin B2 | C20H30O4 | 13367-85-6 | C05954 | 334.2226 | 398.8 | [M]+ |
| Muramic acid | C9H17NO7 | 1114-41-6 | C06470 | 251.0944 | 247.5 | [M]+ |
| Jasmonic acid | C12H18O3 | 59366-47-1 | C08491 | 193.1226 | 532.6 | [M+H-H2O]+ |
| Phenylpyruvic acid | C9H8O3 | 156-06-9 | C00166 | 165.0583 | 132.9 | [M+H]+ |
| Barbituric acid | C4H4N2O3 | 67-52-7 | C00813 | 128.0194 | 44.6 | [M]+ |
| 6-Hydroxyhexanoic acid | C6H12O3 | 1191-25-9 | C06103 | 131.0685 | 216.3 | [M-H]- |
| Pantothenic acid | C9H17NO5 | 79-83-4 | C00864 | 202.1075 | 192.1 | [M+H-H2O]+ |
| 4-Quinolinecarboxylic acid | C10H7NO2 | 486-74-8 | C06414 | 173.0441 | 513.1 | [M]- |
| 3-Benzylmalic acid | C11H12O5 | NA | C20654 | 207.065 | 298.4 | [M+H-H2O]+ |
| Kojic acid | C6H6O4 | 501-30-4 | C14516 | 143.0366 | 223.9 | [M+H]+ |
| Sebacic acid | C10H18O4 | 111-20-6 | C08277 | 185.1186 | 327.2 | [M+H-H2O]+ |
| 2-Hydroxy-3-(4-hydroxyphenyl)propenoic acid | C9H8O4 | 52178-61-7 | C05350 | 181.0528 | 98.7 | [M+H]+ |
| 5-Hydroxypentanoic acid | C5H10O3 | 13392-69-3 | C02804 | 118.065 | 195.6 | [M]+ |
| Stearolic acid | C18H32O2 | 506-24-1 | C08459 | 280.2355 | 430.8 | [M]+ |
| Oleic acid | C18H34O2 | 112-80-1 | C00712 | 265.2523 | 597.6 | [M+H-H2O]+ |
| 11-Dehydro-thromboxane B2 | C20H32O6 | 67910-12-7 | C05964 | 351.2175 | 487.1 | [M+H-H2O]+ |
| 5,6-DHET | C20H34O4 | 213382-49-1 | C14772 | 321.2427 | 532.4 | [M+H-H2O]+ |
| 8,9-DiHETrE | C20H34O4 | 192461-96-4 | C14773 | 321.2426 | 619.6 | [M+H-H2O]+ |
| Prostaglandin H2 | C20H32O5 | 42935-17-1 | C00427 | 353.2367 | 449.7 | [M+H]+ |
| 13,14-Dihydro-15-keto-PGE2 | C20H32O5 | 363-23-5 | C04671 | 335.2216 | 563.3 | [M+H-H2O]+ |
| Suberic acid | C8H14O4 | 505-48-6 | C08278 | 157.0857 | 305.1 | [M+H-H2O]+ |
| 4-Acetylbutyrate | C6H10O3 | 3128-06-1 | C02129 | 129.0542 | 128 | [M-H]- |
| Prostaglandin F2a | C20H34O5 | 551-11-1 | C00639 | 337.2371 | 495 | [M+H-H2O]+ |
| 2-Pyrocatechuic acid | C7H6O4 | 303-38-8 | C00196 | 153.0211 | 150.6 | [M-H]- |
| Gentisic acid | C7H6O4 | 490-79-9 | C00628 | 153.0211 | 161.4 | [M-H]- |
| Hippuric acid | C9H9NO3 | 495-69-2 | C01586 | 179.0602 | 41.4 | [M]+ |
| 2-Keto-glutaramic acid | C5H7NO4 | 18465-19-5 | C00940 | 145.0479 | 133 | [M]+ |
| L-Malic acid | C4H6O5 | 97-67-6 | C00149 | 133.0119 | 49.4 | [M-H]- |
| Bovinic acid | C18H32O2 | 2540-56-9 | C04056 | 279.23 | 639.7 | [M-H]- |
| Norlinolenic acid | C17H28O2 | NA | C16344 | 264.2062 | 236.8 | [M]+ |
| Ketoleucine | C6H10O3 | 816-66-0 | C00233 | 129.0544 | 66 | [M-H]- |
| Dodecanedioic acid | C12H22O4 | 693-23-2 | C02678 | 213.1484 | 546 | [M+H-H2O]+ |
| 4-Hydroxybenzoic acid | C7H6O3 | 99-96-7 | C00156 | 137.0231 | 686.4 | [M-H]- |
| 2-Aminobenzoic acid | C7H7NO2 | 118-92-3 | C00108 | 136.0392 | 209.2 | [M-H]- |
| p-Anisic acid | C8H8O3 | 100-09-4 | C02519 | 151.0387 | 201.7 | [M-H]- |
| α-Ketoisovaleric acid | C5H8O3 | 759-05-7 | C00141 | 117.0556 | 281.5 | [M+H]+ |
| Aminocaproic acid | C6H13NO2 | 60-32-2 | C02378 | 132.1007 | 179.3 | [M+H]+ |
| Pimelic acid | C7H12O4 | 111-16-0 | C02656 | 159.9703 | 33.3 | [M]+ |
| Prostaglandin F2b | C20H34O5 | 4510-16-1 | C02314 | 355.2504 | 518.4 | [M+H]+ |
| Resolvin D2 | C22H32O5 | 82864-77-5 | C18179 | 377.2296 | 486.7 | [M+H]+ |
| 4-Pyridoxic acid | C8H9NO4 | 82-82-6 | C00847 | 182.987 | 143.1 | [M]+ |
| Palmitic acid | C16H32O2 | 57-10-3 | C00249 | 255.2309 | 638.4 | [M-H]- |
| Hydrocinnamic acid | C9H10O2 | 501-52-0 | C05629 | 151.0764 | 306.7 | [M+H]+ |
| 4-Hydroxycinnamic acid | C9H8O3 | 7400-08-0 | C00811 | 146.981 | 650.2 | [M+H-H2O]+ |
| Phosphoglycolic acid | C2H5O6P | 13147-57-4 | C00988 | 156.9907 | 686.5 | [M+H]+ |
| 9(S)-HPOT | C18H30O4 | 111004-08-1 | C16321 | 311.2198 | 466.4 | [M+H]+ |
| Adipic acid | C6H10O4 | 124-04-9 | C06104 | 127.0388 | 105.1 | [M-H2O-H]- |
| Nonadecanoic acid | C19H38O2 | 646-30-0 | C16535 | 297.2419 | 479.6 | [M-H]- |
| Gallic acid | C7H6O5 | 149-91-7 | C01424 | 169.0133 | 131 | [M-H]- |
| Oxalacetic acid | C4H4O5 | 328-42-7 | C00036 | 112.9838 | 252.5 | [M-H2O-H]- |
| Salicyluric acid | C9H9NO4 | 487-54-7 | C07588 | 194.0449 | 90.3 | [M-H]- |
| Guanidoacetic acid | C3H7N3O2 | 352-97-6 | C00581 | 117.0544 | 686.4 | [M]- |
| Citric acid | C6H8O7 | 77-92-9 | C00158 | 191.0188 | 48.5 | [M-H]- |
| Phthalic acid | C8H6O4 | 88-99-3 | C01606 | 165.0409 | 569.3 | [M-H]- |
| 3-Hydroxyphenylacetic acid | C8H8O3 | 621-37-4 | C05593 | 153.0553 | 358.4 | [M+H]+ |
| Citramalic acid | C5H8O5 | 597-44-4 | C00815 | 129.0182 | 193.9 | [M-H2O-H]- |
| Creatine | C4H9N3O2 | 57-00-1 | C00300 | 131.0701 | 93.8 | [M]+ |
| β-Guanidinopropionic acid | C4H9N3O2 | 353-09-3 | C03065 | 131.0701 | 137.1 | [M]+ |
| Pyrroline hydroxycarboxylic acid | C5H7NO3 | 22573-88-2 | C04281 | 130.0493 | 495.9 | [M+H]+ |
| p-Hydroxyphenylacetic acid | C8H8O3 | 156-38-7 | C00642 | 153.0539 | 218.8 | [M+H]+ |
| Erucic acid | C22H42O2 | 112-86-7 | C08316 | 338.3424 | 681.7 | [M]+ |
| all-trans-Retinoic acid | C20H28O2 | 302-79-4 | C00777 | 299.2576 | 553.1 | [M-H]- |
| Uric acid | C5H4N4O3 | 69-93-2 | C00366 | 169.0867 | 250.8 | [M+H]+ |
| Oxoadipic acid | C6H8O5 | 3184-35-8 | C00322 | 158.9778 | 73.7 | [M-H]- |
| 3-(3,4-Dihydroxy-5-methoxy)-2-propenoic acid | C10H10O5 | 1782-55-4 | C05619 | 209.0447 | 244.8 | [M-H]- |
| Shikimic acid | C7H10O5 | 138-59-0 | C00493 | 173.044 | 171.9 | [M-H]- |
| 20-Carboxy-leukotriene B4 | C20H30O6 | 80434-82-8 | C05950 | 367.2102 | 454.7 | [M+H]+ |
| 9,10-DHOME | C18H34O4 | 263399-34-4 | C14828 | 297.2437 | 520.1 | [M+H-H2O]+ |
| Heptanoic acid | C7H14O2 | 111-14-8 | C17714 | 130.0082 | 689.3 | [M]+ |
| Quinic acid | C10H13NO | 77-95-2 | C06746 | 164.1075 | 301.9 | [M+H]+ |
| Pyrrolidonecarboxylic acid | C5H7NO3 | 4042-36-8 | C02237 | 130.0495 | 393.7 | [M+H]+ |
| Urocanic acid | C6H6N2O2 | 104-98-3 | C00785 | 139.0498 | 56.3 | [M+H]+ |
| Sinapic acid | C11H12O5 | 530-59-6 | C00482 | 223.0606 | 352 | [M-H]- |
| Prostaglandin E1 | C20H34O5 | 745-65-3 | C04741 | 337.237 | 454.8 | [M+H-H2O]+ |
| 2-Isopropylmalic acid | C7H12O5 | 3237-44-3 | C02504 | 157.0497 | 197 | [M-H2O-H]- |
| 3,4-Dihydroxyhydrocinnamic acid | C9H10O4 | 1078-61-1 | C10447 | 163.0399 | 292.3 | [M-H2O-H]- |
| Succinic acid | C4H6O4 | 110-15-6 | C00042 | 117.0544 | 66.7 | [M-H]- |
| 9(S)-HPODE | C18H32O4 | 29774-12-7 | C14827 | 295.2266 | 546.3 | [M+H-H2O]+ |
| Nicotinic acid | C6H5NO2 | 59-67-6 | C00253 | 124.039 | 168.8 | [M+H]+ |
| Maleic acid | C4H4O4 | 110-16-7 | C01384 | 115.002 | 192.6 | [M-H]- |
| α-dimorphecolic acid | C18H32O3 | 73543-67-6 | C14767 | 279.2318 | 532.7 | [M+H-H2O]+ |
| 13S-hydroxyoctadecadienoic acid | C18H32O3 | 5204-88-6 | C14762 | 279.2318 | 520.7 | [M+H-H2O]+ |
| Stearic acid | C18H36O2 | 57-11-4 | C01530 | 265.1474 | 651.7 | [M-H2O-H]- |
| Salicylic acid | C7H6O3 | 69-72-7 | C00805 | 137.0247 | 66.6 | [M-H]- |
| Gemfibrozil | C15H22O3 | 25812-30-0 | C07020 | 249.1491 | 499.7 | [M-H]- |
| (2'E,4'Z,7'Z,8E)-Colnelenic acid | C18H28O3 | 52591-16-9 | C16320 | 293.2105 | 533.2 | [M+H]+ |
| Prostaglandin F3a | C20H32O5 | 745-64-2 | C06476 | 335.2216 | 458.8 | [M+H-H2O]+ |
| 2-Hydroxy-6-pentadecylbenzoic acid | C22H36O3 | 16611-84-0 | C10759 | 349.2743 | 527.7 | [M+H]+ |
| Linoleic acid | C18H32O2 | 60-33-3 | C01595 | 281.247 | 459.5 | [M+H]+ |
| Dethiobiotin | C10H18N2O3 | 533-48-2 | C01909 | 213.0397 | 205.7 | [M-H]- |
| Xanthurenic acid | C10H7NO4 | 59-00-7 | C02470 | 204.0296 | 230.4 | [M-H]- |
| 3-Hydroxymethylglutaric acid | C6H10O5 | 503-49-1 | C03761 | 143.0342 | 64.7 | [M-H2O-H]- |
| Prostaglandin D2 | C20H32O5 | 41598-07-6 | C00696 | 353.2328 | 491 | [M+H]+ |
| 3-Hydroxybenzoic acid | C7H6O3 | 99-06-9 | C00587 | 137.0227 | 219.9 | [M-H]- |
| Azelaic acid | C9H16O4 | 123-99-9 | C08261 | 187.0967 | 395.7 | [M-H]- |
| Arachidonic acid | C20H32O2 | 506-32-1 | C00219 | 305.2479 | 545 | [M+H]+ |
| trans-Ferulic acid | C10H10O4 | 537-98-4 | C01494 | 193.0502 | 325.2 | [M-H]- |
| 12-Hydroxydodecanoic acid | C12H24O3 | 505-95-3 | C08317 | 215.0089 | 686.8 | [M-H]- |
| Tartaric acid | C4H6O6 | 87-69-4 | C00898 | 149.0079 | 49.3 | [M-H]- |
| 2-Hydroxy-3-(4-hydroxyphenyl)propanoic acid | C9H10O4 | 6482-98-0 | C03672 | 183.0655 | 275.3 | [M+H]+ |
| γ-Aminobutyric acid | C4H9NO2 | 56-12-2 | C00334 | 104.0712 | 667.9 | [M+H]+ |
| 10-Nitrolinoleic acid | C18H31NO4 | 774603-04-2 | C13800 | 306.2066 | 429.4 | [M-H2O-H]- |
| Guanidinosuccinic acid | C5H9N3O4 | 6133-30-8 | C03139 | 174.9551 | 115.5 | [M]- |
| Farnesoic acid | C15H24O2 | 462-11-3 | C16502 | 237.1851 | 347.5 | [M+H]+ |
| 19(S)-HETE | C20H32O3 | 79551-85-2 | C14749 | 321.2427 | 470 | [M+H]+ |
| Mandelic acid | C8H8O3 | 90-64-2 | C01984 | 153.091 | 338 | [M+H]+ |
| Dodecanoic acid | C12H24O2 | 143-07-7 | C02679 | 199.1695 | 496.4 | [M-H]- |
| 6-Keto-prostaglandin F1a | C20H34O6 | 58962-34-8 | C05961 | 371.2431 | 458.9 | [M+H]+ |
| Mevaldate | C6H10O4 | 541-07-1 | C00772 | 147.0653 | 118.9 | [M+H]+ |
| Oxoglutaric acid | C5H6O5 | 328-50-7 | C00026 | 145.013 | 52.5 | [M-H]- |
| Ortho-Hydroxyphenylacetic acid | C8H8O3 | 614-75-5 | C05852 | 153.0547 | 323.8 | [M+H]+ |
| 8,9-EET | C20H32O3 | 81246-85-7 | C14769 | 321.2426 | 489.7 | [M+H]+ |
| Isonicotinic acid | C6H5NO2 | 55-22-1 | C07446 | 124.0392 | 458.1 | [M+H]+ |
| 9-OxoODE | C18H30O3 | 54232-59-6 | C14766 | 295.2266 | 353.3 | [M+H]+ |
| Gulonic acid | C6H12O7 | 20246-53-1 | C00800 | 197.0811 | 314.8 | [M+H]+ |
| Prostaglandin F1a | C20H36O5 | 745-62-0 | C06475 | 355.2488 | 453.1 | [M-H]- |
| 12,13-DHOME | C18H34O4 | 263399-35-5 | C14829 | 315.2528 | 431.3 | [M+H]+ |
| Caproic acid | C6H12O2 | 142-62-1 | C01585 | 114.9329 | 666.7 | [M-H]- |
| α-Oxo-benzeneacetic acid | C8H6O3 | 611-73-4 | C02137 | 149.0245 | 263.9 | [M-H]- |
| Arachidic acid | C20H40O2 | 506-30-9 | C06425 | 311.2953 | 680 | [M-H]- |
| 3-Methylthiopropionic acid | C4H8O2S | 646-01-5 | C08276 | 119.0489 | 140.9 | [M-H]- |
| Stearidonic acid | C18H28O2 | 20290-75-9 | C16300 | 277.2163 | 601.2 | [M+H]+ |
| (6Z)-Octadecenoic acid | C18H34O2 | 593-39-5 | C08363 | 281.2487 | 655.6 | [M-H]- |
| 9(S)-HOT | C18H30O3 | 89886-42-0 | C16326 | 295.2267 | 369.3 | [M+H]+ |
| 13-L-Hydroperoxylinoleic acid | C18H32O4 | 33964-75-9 | C04717 | 313.2373 | 451.9 | [M+H]+ |
| Phenylacetic acid | C8H8O2 | 103-82-2 | C07086 | 137.0597 | 273.5 | [M+H]+ |
| Caprylic acid | C8H16O2 | 124-07-2 | C06423 | 124.99 | 66.7 | [M-H2O-H]- |
| Tetrahydrodipicolinate | C7H9NO4 | 2353-17-5 | C03972 | 172.0604 | 114.4 | [M+H]+ |
| 3,4-Dihydroxymandelic acid | C8H8O5 | 14883-87-5 | C05580 | 184.0324 | 222.7 | [M]- |
| 11,12-DiHETrE | C20H34O4 | 192461-95-3 | C14774 | 339.253 | 532.6 | [M+H]+ |
| 14,15-DiHETrE | C20H34O4 | 77667-09-5 | C14775 | 339.253 | 502.4 | [M+H]+ |
| (+)-7-Isojasmonic acid | C12H18O3 | 62653-85-4 | C16317 | 211.1333 | 463.3 | [M+H]+ |
| Fumaric acid | C4H4O4 | 110-17-8 | C00122 | 115.0386 | 52.5 | [M-H]- |
| Traumatic Acid | C12H20O4 | 6402-36-4 | C16308 | 229.1435 | 348.7 | [M+H]+ |
| 10-Hydroxydecanoic acid | C10H20O3 | 1679-53-4 | C02774 | 187.1332 | 294.2 | [M-H]- |
| 13(S)-HpOTrE | C18H30O4 | 67597-26-6 | C04785 | 311.2218 | 418.8 | [M+H]+ |
| **Carbohydrates and derivatives** | | | | | | |
| 3,6-Anhydroglucose | C6H10O5 | 7625-23-2 | C06478 | 145.0496 | 576.9 | [M+H-H2O]+ |
| L-Iditol | C6H14O6 | 488-45-9 | C01507 | 181.0673 | 499.2 | [M-H]- |
| D-Iditol | C6H14O6 | 25878-23-3 | C01489 | 181.0673 | 545.6 | [M-H]- |
| Fucose 1-phosphate | C6H13O8P | 16562-58-6 | C02985 | 243.0327 | 381.7 | [M-H]- |
| N-Acetyl-D-glucosamine | C8H15NO6 | 7512-17-6 | C00140 | 204.0866 | 55 | [M+H-H2O]+ |
| 1-O-Galloyl-β-D-glucose | C13H16O10 | 554-37-0 | C01158 | 313.05 | 59 | [M-H2O-H]- |
| D-Fructose | C6H12O6 | 57-48-7 | C00095 | 161.0421 | 128.4 | [M-H2O-H]- |
| D-Galactose | C6H12O6 | 59-23-4 | C00124 | 161.0421 | 375.8 | [M-H2O-H]- |
| Fructose-1P | C6H12O6 | 57-48-7 | C10906 | 161.0421 | 335.5 | [M-H2O-H]- |
| D-Mannose | C6H12O6 | 3458-28-4 | C00159 | 161.0421 | 157.5 | [M-H2O-H]- |
| Galactitol | C6H14O6 | 608-66-2 | C01697 | 183.0896 | 96.2 | [M+H]+ |
| D-Glucose | C6H12O6 | 50-99-7 | C00031 | 161.0422 | 414.7 | [M-H2O-H]- |
| Ribitol | C5H12O5 | 488-81-3 | C00474 | 152.0711 | 244.8 | [M]+ |
| D-Xylose | C5H10O5 | 58-86-6 | C00181 | 131.0322 | 178.5 | [M-H2O-H]- |
| α-D-Mannose | C6H12O6 | 3458-28-4 | C00936 | 161.0423 | 177 | [M-H2O-H]- |
| α-D-Glucose | C6H12O6 | 492-62-6 | C00267 | 161.0423 | 252.7 | [M-H2O-H]- |
| L-Ribulose | C5H10O5 | 2042-27-5 | C00310 | 131.0323 | 248.4 | [M-H2O-H]- |
| D-Glycero-D-galacto-heptitol | C7H16O7 | 527-06-0 | C08255 | 212.0928 | 261.5 | [M]+ |
| 1,5-Anhydrosorbitol | C6H12O5 | 154-58-5 | C07326 | 163.0589 | 616.4 | [M-H]- |
| Sedoheptulose | C7H14O7 | 3019-74-7 | C02076 | 209.0646 | 595.1 | [M-H]- |
| D-Xylitol | C5H12O5 | 87-99-0 | C00379 | 151.0589 | 403.4 | [M-H]- |
| D-Arabitol | C5H12O5 | 7643-75-6 | C01904 | 151.06 | 428.6 | [M-H]- |
| Erythritol | C4H10O4 | 149-32-6 | C00503 | 121.0276 | 278.7 | [M-H]- |
| Sorbitol | C6H14O6 | 50-70-4 | C00794 | 181.0147 | 66.6 | [M-H]- |
| L-Erythrulose | C4H8O4 | 533-50-6 | C02045 | 101.0234 | 203.3 | [M-H2O-H]- |
| Mannitol | C6H14O6 | 69-65-8 | C00392 | 163.0599 | 487.6 | [M-H2O-H]- |
| 6-Tuliposide A | C11H18O8 | 19870-31-6 | C20577 | 277.092 | 120.7 | [M-H]- |
| L-Fucose | C6H12O5 | 2438-80-4 | C00507 | 164.068 | 44.6 | [M]+ |
| 6-Tuliposide B | C11H18O9 | 244105-18-8 | C21186 | 293.0871 | 223.9 | [M-H]- |
| Allose | C6H12O6 | 6038-51-3 | C01487 | 181.0144 | 685.3 | [M+H]+ |
| D-Ribose | C5H10O5 | 613-83-2 | C00121 | 151.0352 | 44.6 | [M+H]+ |
| 1-O-Feruloyl-β-D-glucose | C16H20O9 | NA | C17759 | 355.1029 | 189 | [M-H]- |
| Gluconic acid | C6H12O7 | 526-95-4 | C00257 | 197.0813 | 250.2 | [M+H]+ |
| trans-Cinnamoyl β-D-glucoside | C15H18O7 | 13080-39-2 | C04164 | 309.0976 | 234.1 | [M-H]- |
| 6-Acetyl-D-glucose | C8H14O7 | 7286-45-5 | C02655 | 203.0554 | 103.2 | [M-H2O-H]- |
| D-Arabinose | C5H10O5 | 10323-20-3 | C00216 | 149.9933 | 514.6 | [M]- |
| Glucosamine | C6H13NO5 | 3416-24-8 | C00329 | 180.0867 | 49.4 | [M+H]+ |
| L-Arabinose | C5H10O5 | 5328-37-0 | C00259 | 149.9932 | 580.8 | [M]- |
| Trehalose | C12H22O11 | 99-20-7 | C01083 | 323.0978 | 108.7 | [M-H2O-H]- |
| Coniferin | C16H22O8 | 531-29-3 | C00761 | 341.1242 | 232.9 | [M-H]- |
| Fructose 1,6-bisphosphate | C6H14O12P2 | 488-69-7 | C00354 | 339.1998 | 458.1 | [M-H]- |
| **Amino acid and derivatives** | | | | | | |
| N-Acetyl-L-glutamate 5-semialdehyde | C7H11NO4 | 13074-21-0 | C01250 | 156.0656 | 168.2 | [M+H-H2O]+ |
| 6-Aminopenicillanic acid | C8H12N2O3S | 551-16-6 | C02954 | 215.0552 | 123.4 | [M-H]- |
| N-methyl-L-glutamic Acid | C6H11NO4 | 35989-16-3 | C01046 | 144.0648 | 94.3 | [M+H-H2O]+ |
| L-Arogenate | C10H13NO5 | 53078-86-7 | C00826 | 228.0906 | 139.2 | [M+H]+ |
| L-Histidine | C6H9N3O2 | 71-00-1 | C00135 | 156.0423 | 49.2 | [M+H]+ |
| Pyroglutamic acid | C5H7NO3 | 98-79-3 | C01879 | 128.0339 | 193.1 | [M-H]- |
| L-Valine | C5H11NO2 | 72-18-4 | C00183 | 118.0875 | 275.8 | [M+H]+ |
| 5-Aminopentanoic acid | C5H11NO2 | 660-88-8 | C00431 | 118.0875 | 172.3 | [M+H]+ |
| Argininosuccinic acid | C10H18N4O6 | 2387-71-5 | C03406 | 271.1018 | 431 | [M-H2O-H]- |
| L-Proline | C5H9NO2 | 344-25-2 | C00148 | 116.0716 | 541.9 | [M+H]+ |
| 3-Methyl-L-tyrosine | C10H13NO3 | 2370-57-2 | C20800 | 196.0952 | 234.1 | [M+H]+ |
| Se-Methylselenocysteine | C4H9NO2Se | 26046-90-2 | C05689 | 182.984 | 314.2 | [M]+ |
| Folic acid | C19H19N7O6 | 59-30-3 | C00504 | 441.2498 | 456.4 | [M]- |
| 3,5-Diiodo-L-tyrosine | C9H9I2NO3 | 300-39-0 | C01060 | 433.1005 | 524.1 | [M]- |
| L-Isoleucine | C6H13NO2 | 73-32-5 | C00407 | 132.1013 | 81.6 | [M+H]+ |
| 4-Acetamido-2-aminobutanoic acid | C6H12N2O3 | 1190-46-1 | C06442 | 160.0974 | 54.3 | [M]+ |
| Betaine | C5H11NO2 | 107-43-7 | C00719 | 116.9269 | 605.4 | [M]- |
| Dimethylglycine | C4H9NO2 | 1118-68-9 | C01026 | 104.0709 | 51.9 | [M+H]+ |
| N,N-Dimethylhistidine | C8H13N3O2 | 24940-57-6 | C04259 | 183.1022 | 333.9 | [M]+ |
| L-Phenylalanine | C9H11NO2 | 63-91-2 | C00079 | 166.0859 | 345.3 | [M+H]+ |
| γ-Glutamylcysteine | C8H14N2O5S | 636-58-8 | C00669 | 250.144 | 527.8 | [M]- |
| L-Lysine | C6H14N2O2 | 56-87-1 | C00047 | 147.113 | 47 | [M+H]+ |
| N6-Acetyl-L-lysine | C8H16N2O3 | 692-04-6 | C02727 | 189.1236 | 77.5 | [M+H]+ |
| L-Targinine | C7H16N4O2 | 17035-90-4 | C03884 | 189.1348 | 47.2 | [M+H]+ |
| L-Arginine | C6H14N4O2 | 74-79-3 | C00062 | 175.1191 | 47.4 | [M+H]+ |
| (S)-β-Tyrosine | C9H11NO3 | NA | C21308 | 182.0811 | 273.3 | [M+H]+ |
| β-Tyrosine | C9H11NO3 | NA | C04368 | 182.0811 | 210.4 | [M+H]+ |
| N-Acetyl-L-phenylalanine | C11H13NO3 | 2018-61-3 | C03519 | 206.0816 | 186.3 | [M-H]- |
| 3-Aminoisobutanoic acid | C4H9NO2 | 144-90-1 | C05145 | 104.0711 | 103 | [M+H]+ |
| L-2,4-diaminobutyric acid | C4H10N2O2 | 1758-80-1 | C03283 | 118.0652 | 513.1 | [M]+ |
| Selenocysteine | C3H7NO2Se | 3614-08-2 | C05688 | 169.9772 | 65.5 | [M+H]+ |
| N-Acetylleucine | C8H15NO3 | 1188-21-2 | C02710 | 172.0966 | 126.6 | [M-H]- |
| L-Glutamine | C5H10N2O3 | 5959-95-5 | C00064 | 146.0813 | 202.2 | [M]+ |
| **Aromatic compounds** | | | | | | |
| 3,5-Dihydroxyphenylglyoxylate | C8H6O5 | NA | C12325 | 165.0183 | 314.1 | [M+H-H2O]+ |
| Ketoprofen | C16H14O3 | 22071-15-4 | C01716 | 253.0931 | 463.7 | [M-H]- |
| Benzoate | C7H6O2 | 65-85-0 | C00180 | 123.0469 | 237 | [M+H]+ |
| trans,trans-1,4-Diphenyl-1,3-butadiene | C16H14 | 538-81-8 | C14761 | 207.1131 | 207.8 | [M+H]+ |
| Methoxamine | C11H17NO3 | 390-28-3 | C07513 | 194.1168 | 362.8 | [M+H-H2O]+ |
| Bz-Arg-OEt | C15H22N4O3 | NA | C01670 | 289.1644 | 356.4 | [M+H-H2O]+ |
| Sinapoyl aldehyde | C11H12O4 | 4206-58-0 | C05610 | 209.0826 | 286.9 | [M+H]+ |
| 1,2,3-Trihydroxybenzene | C6H6O3 | 87-66-1 | C01108 | 127.0402 | 257.4 | [M+H]+ |
| Dhurrin | C14H17NO7 | 499-20-7 | C05143 | 311.0986 | 132.8 | [M]- |
| 2-Phenylacetamide | C8H9NO | 103-81-1 | C02505 | 136.0764 | 280.1 | [M+H]+ |
| N-Acetylanthranilate | C9H9NO3 | 89-52-1 | C06332 | 178.0501 | 165.6 | [M-H]- |
| Rutin | C27H30O16 | 207671-50-9 | C05625 | 610.1882 | 565.9 | [M]+ |
| Phenylethylamine | C8H11N | 64-04-0 | C05332 | 121.0276 | 489.7 | [M]- |
| Tyramine | C8H11NO | 51-67-2 | C00483 | 138.0911 | 231.2 | [M+H]+ |
| trans-4-Hydroxystilbene | C14H12O | 6554-98-9 | C14763 | 179.0817 | 89.2 | [M+H-H2O]+ |
| Tyrosol | C8H10O2 | 501-94-0 | C06044 | 139.0752 | 321.3 | [M+H]+ |
| 3,5-Dihydroxy-phenylglycine | C8H9NO4 | 146255-66-5 | C12026 | 184.0607 | 232.1 | [M+H]+ |
| Prephenate | C10H10O6 | 126-49-8 | C00254 | 207.0291 | 235.1 | [M-H2O-H]- |
| 2,6-Dimethylaniline | C8H11N | 87-62-7 | C11004 | 122.0965 | 125.1 | [M+H]+ |
| N-Methyltyramine | C9H13NO | 370-98-9 | C02442 | 152.1069 | 338.4 | [M+H]+ |
| trans-Isoasarone | C12H16O3 | 2883-98-9 | C17846 | 209.1173 | 379.4 | [M+H]+ |
| Antiarol | C9H12O4 | 642-71-7 | C10765 | 185.0808 | 330.5 | [M+H]+ |
| Diglycidyl resorcinol ether | C12H14O4 | 101-90-6 | C19228 | 223.0965 | 282.2 | [M+H]+ |
| Puerarin | C21H20O9 | 3681-99-0 | C10524 | 415.1034 | 220.4 | [M-H]- |
| **Esters** | | | | | | |
| Ecgonine methyl ester | C10H17NO3 | 7143-09-1 | C12448 | 182.1177 | 185.6 | [M+H-H2O]+ |
| (4Z,7Z,10Z,13Z,16Z,19Z)-Docosahexaenoic acid ethyl ester | C24H36O2 | 81926-94-5 | C16185 | 356.2794 | 376.1 | [M]+ |
| Lovastatin | C24H36O5 | 75330-75-5 | C07074 | 387.257 | 418.2 | [M+H-H2O]+ |
| Phosphonoacetate | C2H5O5P | 4408-78-0 | C05682 | 140.0698 | 167.9 | [M]+ |
| Succinic acid semialdehyde | C4H6O3 | 692-29-5 | C00232 | 101.0235 | 688.7 | [M-H]- |
| Phenyllactate | C9H10O3 | 7326-19-4 | C05607 | 165.0547 | 128.7 | [M-H]- |
| (S)-5-Amino-3-oxohexanoate | C6H11NO3 | 19355-90-9 | C03656 | 146.0821 | 411.6 | [M+H]+ |
| 3-Amino-4-hydroxybenzoate | C7H7NO3 | 1571-72-8 | C12115 | 154.0496 | 76 | [M+H]+ |
| Dihomo-γ-linolenate | C20H34O2 | 1783-84-2 | C03242 | 307.2627 | 543.9 | [M+H]+ |
| (R)-Pantolactone | C6H10O3 | 599-04-2 | C01012 | 131.5334 | 199.1 | [M+H]+ |
| Menthyl pyrrolidone carboxylate | C16H30O6 | 52528-10-6 | C03962 | 319.2118 | 401.1 | [M+H]+ |
| Acetylphosphate | C2H5O5P | 590-54-5 | C00227 | 139.988 | 685.7 | [M]+ |
| Malonate | C3H4O4 | 141-82-2 | C00383 | 104.0708 | 121 | [M]+ |
| Methyl jasmonate | C13H20O3 | 1211-29-6 | C11512 | 225.1486 | 454.1 | [M+H]+ |
| Oleoylethanolamide | C20H39NO2 | 111-58-0 | C20792 | 326.3055 | 578.4 | [M+H]+ |
| 2-Keto-6-acetamidocaproate | C8H13NO4 | 59403-50-8 | C05548 | 188.0918 | 153.6 | [M+H]+ |
| Neocnidilide | C12H18O2 | 4567-33-3 | C17002 | 195.138 | 546 | [M+H]+ |
| 9,10-Dihydroxystearate | C18H36O4 | NA | C19622 | 299.2584 | 459.6 | [M+H-H2O]+ |
| Phenyl acetate | C8H8O2 | 122-79-2 | C00548 | 134.8938 | 644.5 | [M-H]- |
| 3-Dehydroshikimate | C7H8O5 | 2922-42-1 | C02637 | 171.0287 | 52.1 | [M-H]- |
| Caffeate | C9H8O4 | 501-16-6 | C01197 | 179.0351 | 201.3 | [M-H]- |
| trans-Cinnamate | C9H8O2 | 140-10-3 | C00423 | 146.9648 | 423.6 | [M-H]- |
| Quinate | C7H12O6 | 77-95-2 | C00296 | 191.0553 | 192.8 | [M-H]- |
| Oxalureate | C3H4N2O4 | 585-05-7 | C00802 | 133.0319 | 222.8 | [M+H]+ |
| **Aldehydes and ketones** | | | | | | |
| Hecogenin | C27H42O4 | 467-55-0 | C08902 | 413.2899 | 522.9 | [M+H-H2O]+ |
| 11α,17β-Dihydroxyandrost-4-en-3-one | C19H28O3 | 426212 | C15306 | 304.2118 | 403.9 | [M]+ |
| 4-Hydroxybenzaldehyde | C7H6O2 | 123-08-0 | C00633 | 123.0456 | 333.3 | [M+H]+ |
| Phenylacetaldehyde | C8H8O | 122-78-1 | C00601 | 121.0658 | 487.7 | [M+H]+ |
| 3-Dehydro-2-deoxyecdysone | C27H42O5 | NA | C16497 | 447.3138 | 538.4 | [M+H]+ |
| Xanthoxylin | C10H12O4 | 90-24-4 | C10726 | 195.0653 | 249.6 | [M-H]- |
| L-Glutamic γ-semialdehyde | C5H9NO3 | 496-92-4 | C01165 | 132.0648 | 52.1 | [M+H]+ |
| Betaine aldehyde | C5H12NO | 7418-61-3 | C00576 | 102.0915 | 135.7 | [M]+ |
| Biochanin A | C16H12O5 | 491-80-5 | C00814 | 283.1691 | 527.4 | [M-H]- |
| 4-Methylbenzaldehyde | C8H8O | 104-87-0 | C06758 | 121.0644 | 524.5 | [M+H]+ |
| Octanal | C8H16O | 124-13-0 | C01545 | 129.1278 | 544.4 | [M+H]+ |
| 2-Heptanone | C7H14O | 110-43-0 | C08380 | 113.9644 | 108.9 | [M]+ |
| (3Z,6Z)-3,6-Nonadienal | C9H14O | 21944-83-2 | C16323 | 139.1121 | 450.1 | [M+H]+ |
| Ononin | C22H22O9 | 486-62-4 | C10509 | 431.1346 | 296.8 | [M+H]+ |
| 9-cis-Retinal | C20H28O | 514-85-2 | C16681 | 285.2056 | 532.6 | [M+H]+ |
| 4-Hydroxyphenylacetaldehyde | C8H8O2 | 7339-87-9 | C03765 | 136.0613 | 319.4 | [M]+ |
| Cortolone | C21H34O5 | 516-42-7 | C05481 | 367.2476 | 368.3 | [M+H]+ |
| Genistein | C15H10O5 | 446-72-0 | C06563 | 271.2271 | 498.4 | [M+H]+ |
| 2-trans,6-trans-Farnesal | C15H24O | 502-67-0 | C03461 | 221.1901 | 473.1 | [M+H]+ |
| (R)-Methylmalonate semialdehyde | C4H6O3 | MA | C21030 | 102.0344 | 45 | [M]+ |
| 3-Oxo-5β-steroid | C19H30O | NA | C02797 | 257.2223 | 331.8 | [M+H-H2O]+ |
| **Alcohols** | | | | | | |
| L-Histidinol | C6H11N3O | 4836-52-6 | C00860 | 124.0863 | 586.5 | [M+H-H2O]+ |
| Miglitol | C8H17NO5 | 72432-03-2 | C07708 | 190.1077 | 179.8 | [M+H-H2O]+ |
| 1-Arachidonoylglycerol | C23H38O4 | 124511-15-5 | C13857 | 378.2856 | 465.6 | [M]+ |
| Curcumenol | C15H22O2 | 19431-84-6 | C16942 | 217.1591 | 461.2 | [M+H-H2O]+ |
| 2,2'-Bisphenol F | C13H12O2 | 2467-02-9 | C14747 | 201.0945 | 278 | [M+H]+ |
| Lariciresinol | C20H24O6 | 27003-73-2 | C10646 | 361.1592 | 85.1 | [M+H]+ |
| Ergosta-5,7,22,24(28)-tetraen-3β-ol | C28H42O | 29560-24-5 | C05440 | 394.3292 | 512.4 | [M]+ |
| Zeranol | C18H26O5 | 26538-44-3 | C14752 | 305.1741 | 462.2 | [M+H-H2O]+ |
| 2,3-Butanediol | C4H10O2S2 | 513-85-9 | C00265 | 154.9888 | 237 | [M+H]+ |
| trans-1,2-Cyclohexanediol | C6H12O2 | 1460-57-7 | C03739 | 115.9193 | 666.4 | [M]- |
| Sphinganine | C18H39NO2 | 764-22-7 | C00836 | 284.2947 | 649.6 | [M+H-H2O]+ |
| Retinol | C20H30O | 68-26-8 | C00473 | 285.0644 | 401.7 | [M-H]- |
| Misoprostol | C21H36O5 | 59122-46-2 | C07227 | 369.2629 | 484.4 | [M+H]+ |
| 4-Methylbenzyl alcohol | C8H10O | 589-18-4 | C06757 | 123.0803 | 362.5 | [M+H]+ |
| (S)-1-Phenylethanol | C8H10O | 1445-91-6 | C11348 | 123.0439 | 292.7 | [M+H]+ |
| 9-cis-Retinol | C20H30O | 22737-97-9 | C16682 | 286.2015 | 407.9 | [M]+ |
| Sclareol | C20H36O2 | 515-03-7 | C09183 | 309.2786 | 544.4 | [M+H]+ |
| Perillyl alcohol | C10H16O | 536-59-4 | C02452 | 135.1168 | 434.1 | [M+H-H2O]+ |
| Abscisic alcohol | C15H22O3 | 113472-20-1 | C13456 | 251.1644 | 487.6 | [M+H]+ |
| Sphingosine | C18H37NO2 | 123-78-4 | C00319 | 300.29 | 416.7 | [M+H]+ |
| **Sterols** | | | | | | |
| 3a-Hydroxy-5b-pregnane-20-one | C21H34O2 | 128-20-1 | C05480 | 318.2635 | 472.2 | [M]+ |
| Cortisone | C21H28O5 | 53-06-5 | C00762 | 360.2014 | 321 | [M]+ |
| Cholesterol sulfate | C27H46O4S | 1256-86-6 | C18043 | 466.3207 | 508.3 | [M]+ |
| Calcitriol | C27H44O3 | 32222-06-3 | C01673 | 416.3361 | 551.6 | [M]+ |
| Stigmasterol | C29H48O | 83-48-7 | C05442 | 412.3773 | 445.5 | [M]+ |
| 17α,21-Dihydroxypregnenolone | C21H32O4 | 1167-48-2 | C05487 | 348.2352 | 422.1 | [M]+ |
| 6β-Hydroxytestosterone | C19H28O3 | 62-99-7 | C14497 | 303.1995 | 461.8 | [M-H]- |
| Allocholic acid | C24H40O5 | 2464-18-8 | C00695 | 409.2918 | 545.5 | [M+H]+ |
| Ergocalciferol | C28H44O | 50-14-6 | C05441 | 397.3408 | 490.7 | [M+H]+ |
| Aldosterone | C21H28O5 | 52-39-1 | C01780 | 361.2207 | 523.7 | [M+H]+ |
| Dihydrocortisol | C21H32O5 | 1482-50-4 | C05471 | 365.2303 | 471.7 | [M+H]+ |
| 3a,7a-Dihydroxy-5b-cholestane | C27H48O2 | 3862-26-8 | C05452 | 404.3358 | 562.7 | [M]+ |
| Corticosterone | C21H30O4 | 50-22-6 | C02140 | 346.3316 | 409.8 | [M]+ |
| Medroxyprogesterone | C22H32O3 | 520-85-4 | C07119 | 345.2395 | 590.7 | [M+H]+ |
| Dehydroepiandrosterone | C19H28O2 | 53-43-0 | C01227 | 269.2118 | 505.8 | [M-H2O-H]- |
| **Phenols** | | | | | | |
| Dopamine | C8H11NO2 | 62-31-7 | C03758 | 136.0756 | 248 | [M+H-H2O]+ |
| Phenylephrine | C9H13NO2 | 59-42-7 | C07441 | 150.0916 | 345.5 | [M+H-H2O]+ |
| Metanephrine | C10H15NO3 | 5001-33-2 | C05588 | 180.1018 | 226.2 | [M+H-H2O]+ |
| Epinephrine | C9H13NO3 | 51-43-4 | C00788 | 166.0854 | 249.5 | [M+H-H2O]+ |
| 3-Methoxy-4-hydroxyphenylglycolaldehyde | C9H10O4 | 17592-23-3 | C05583 | 183.0631 | 203.5 | [M+H]+ |
| Maltol | C6H6O3 | 118-71-8 | C11918 | 125.0231 | 201.3 | [M-H]- |
| 4-Nitrophenol | C6H5NO3 | 100-02-7 | C00870 | 138.0183 | 314.3 | [M-H]- |
| Acetaminophen | C8H9NO2 | 103-90-2 | C06804 | 150.0549 | 257.5 | [M-H]- |
| Sinapyl alcohol | C11H14O4 | 537-33-7 | C02325 | 193.0876 | 345.8 | [M+H-H2O]+ |
| p-Octopamine | C8H11NO2 | 104-14-3 | C04227 | 154.0856 | 269.9 | [M+H]+ |
| Norepinephrine | C8H11NO3 | 51-41-2 | C00547 | 150.055 | 244.8 | [M-H2O-H]- |
| Hydroquinone | C6H6O2 | 123-31-9 | C00530 | 111.0439 | 101.3 | [M+H]+ |
| m-Cresol | C7H8O | 108-39-4 | C01467 | 109.1018 | 430.8 | [M+H]+ |
| Catechol | C6H6O2 | 120-80-9 | C00090 | 111.0921 | 79.5 | [M+H]+ |
| **Others** | | | | | | |
| Niacinamide | C6H6N2O | 98-92-0 | C00153 | 123.0477 | 58.4 | [M+H]+ |
| 1-Deoxynojirimycin | C6H13NO4 | 19130-96-2 | C16843 | 146.081 | 326.7 | [M+H-H2O]+ |
| Cannabielsoin | C21H30O3 | 52025-76-0 | C20218 | 330.2279 | 318.3 | [M]+ |
| Pyridoxine | C8H11NO3 | 65-23-6 | C00314 | 152.0701 | 282.7 | [M+H-H2O]+ |
| Aesculin | C15H16O9 | 531-75-9 | C09264 | 339.0641 | 580.8 | [M-H]- |
| Serotonin | C10H12N2O | 50-67-9 | C00780 | 176.0911 | 57.8 | [M]+ |
| Kalihinol A | C22H33ClN2O2 | 91294-83-6 | C17004 | 393.2227 | 458.6 | [M+H]+ |
| Gitogenin | C27H44O4 | 511-96-6 | C08899 | 432.332 | 528.2 | [M]+ |
| Paraxanthine | C7H8N4O2 | 611-59-6 | C13747 | 163.0602 | 156.3 | [M+H-H2O]+ |
| Andrographolide | C20H30O5 | 5508-58-7 | C20214 | 333.2067 | 538.4 | [M+H-H2O]+ |
| Digitogenin | C27H44O5 | 511-34-2 | C08896 | 448.3248 | 446.2 | [M]+ |
| Thymine | C5H6N2O2 | 65-71-4 | C00178 | 125.0342 | 685.8 | [M-H]- |
| Cerulenin | C12H17NO3 | 17397-89-6 | C12058 | 224.1304 | 351.9 | [M+H]+ |
| Myriocin | C21H39NO6 | 35891-70-4 | C19914 | 384.2665 | 530.4 | [M+H-H2O]+ |
| γ-Glutamyl-β-cyanoalanine | C9H13N3O5 | 16051-95-9 | C05711 | 243.0834 | 221.7 | [M]+ |
| Adenine | C5H5N5 | 73-24-5 | C00147 | 134.0451 | 381.6 | [M-H]- |
| 2,6-Diamino-4-hydroxy-5-N-methylformamidopyrimidine | C6H9N5O2 | 77440-13-2 | C04744 | 183.0769 | 251.8 | [M]+ |
| Cortisol 21-acetate | C23H32O6 | 50-03-3 | C02821 | 405.23 | 517.5 | [M+H]+ |
| 4-Hydroxycinnamoylmethane | C10H10O2 | 3160-35-8 | C12088 | 162.0772 | 49.1 | [M]+ |
| Methyl 5-hydroxy-2-benzimidazole carbamate | C9H9N3O3 | 22769-68-2 | C10902 | 207.0656 | 235.7 | [M]- |
| Dihydrouracil | C4H6N2O2 | 504-07-4 | C00429 | 112.9838 | 137.4 | [M-H]- |
| Acetylcholine chloride | C7H16NO2. Cl | 60-31-1 | C08201 | 181.0862 | 166 | [M]+ |
| 3-Indoleacetonitrile | C10H8N2 | 771-51-7 | C02938 | 157.0869 | 204.7 | [M+H]+ |
| γ-Glutamyl-β-aminopropiononitrile | C8H13N3O3 | 539-95-7 | C06114 | 199.0965 | 243.2 | [M]+ |
| Lanosterin | C30H50O | 79-63-0 | C01724 | 425.2565 | 494.2 | [M-H]- |
| Guanosine | C10H13N5O5 | 118-00-3 | C00387 | 283.263 | 640.4 | [M]- |
| 5'-Methylthioadenosine | C11H15N5O3S | 2457-80-9 | C00170 | 297.2424 | 529.9 | [M]- |
| Dihydrothymine | C5H8N2O2 | 696-04-8 | C00906 | 128.0714 | 443.2 | [M]+ |
| Spermidine | C7H19N3 | 124-20-9 | C00315 | 145.0506 | 639 | [M]+ |
| (S)-2-Phenyloxirane | C8H8O | 20780-54-5 | C20782 | 121.0644 | 512 | [M+H]+ |
| Aminohydroquinone | C6H7NO2 | 20734-68-3 | C14604 | 126.0546 | 55.5 | [M+H]+ |
| Anabasine | C10H14N2 | 13078-04-1 | C06180 | 144.9829 | 632.3 | [M+H-H2O]+ |
| Caffeine | C8H10N4O2 | 58-08-2 | C07481 | 194.0809 | 230.5 | [M]- |
| (R)-5,6-Dihydrothymine | C5H8N2O2 | 86387-01-1 | C21028 | 128.0703 | 416 | [M]+ |
| 2,3-Dinor-8-iso prostaglandin F1α | C18H32O5 | 221664-04-6 | C14795 | 329.2315 | 376.8 | [M+H]+ |
| Sodium deoxycholate | C24H39O4. Na | 302-95-4 | C11171 | 414.3211 | 544.9 | [M]+ |
| 5-(2-Hydroxyethyl)-4-methylthiazole | C6H9NOS | 137-00-8 | C04294 | 144.0481 | 92 | [M+H]+ |
| 2,3-Dinor-8-iso prostaglandin F2α | C18H30O5 | 221664-05-7 | C14794 | 327.2172 | 408.4 | [M+H]+ |
| 3-Methyladenine | C6H7N5 | 5142-23-4 | C00913 | 149.0084 | 524.3 | [M]- |
| Oleamide | C18H35NO | 301-02-0 | C19670 | 282.2796 | 549.1 | [M+H]+ |
| Pyridoxamine | C8H12N2O2 | 85-87-0 | C00534 | 169.0974 | 338 | [M+H]+ |
| Tridemorph | C19H39NO | 81412-43-3 | C11285 | 298.3101 | 677.3 | [M+H]+ |
| Creatinine | C4H7N3O | 60-27-5 | C00791 | 112.9845 | 68.4 | [M]- |
| Triethylamine | C6H15N | 121-44-8 | C14691 | 102.034 | 62.8 | [M+H]+ |
| Cantharidin | C10H12O4 | 56-25-7 | C16778 | 197.0812 | 302.9 | [M+H]+ |
| Palmitoylethanolamide | C18H37NO2 | 544-31-0 | C16512 | 300.2899 | 566.9 | [M+H]+ |
| 12-Keto-tetrahydro-leukotriene B4 | C20H32O4 | 71160-24-2 | C02165 | 336.3096 | 569.4 | [M]+ |
| Isoelemicin | C12H16O3 | 5273-85-8 | C16975 | 209.1173 | 361.4 | [M+H]+ |
| 4,5,6,7-Tetrahydroisoxazolo(5,4-c)pyridin-3-ol | C6H8N2O2 | 64603-91-4 | C13693 | 141.066 | 55.5 | [M+H]+ |
| Uracil | C4H4N2O2 | 66-22-8 | C00106 | 111.0187 | 463.7 | [M-H]- |

**Table S4** Determination of minimum inhibitory concentration

| Concentration (mg/L) | Con | NACs | Lactic acids |
| --- | --- | --- | --- |
| 10000 | ○ | × | × |
| 5000 | ○ | × | × |
| 2500 | ○ | × | × |
| 1250 | ○ | × | × |
| 625 | ○ | × | × |
| 312.50 | ○ | × | × |
| 156.25 | ○ | × | ○ |
| 78.13 | ○ | ○ | ○ |
| 39.07 | ○ | ○ | ○ |
| 19.54 | ○ | ○ | ○ |

The symbol "○" is used to denote turbidity that is visible to the naked eye, whereas "×" indicates the absence of such turbidity.

**SUPPLEMENTARY FIGURES**


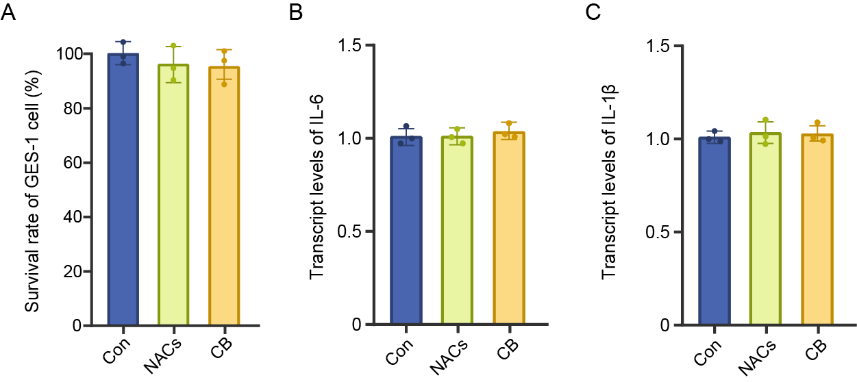


**Figure S1** Effect of CB and NACs alone on GES-1 cell viability and the mRNA expression of pro-inflammatory cytokines. (A) Survival rate of GES-1 cells. (B) The transcript levels of IL-6. (C) The transcript levels of IL-1β. Con group, non-treatment group; The CB group, GES-1 cell treated with 1% ethanol concentration of CB; The NACs group, GES-1 cell treated with 24.9 mg/L of NACs.


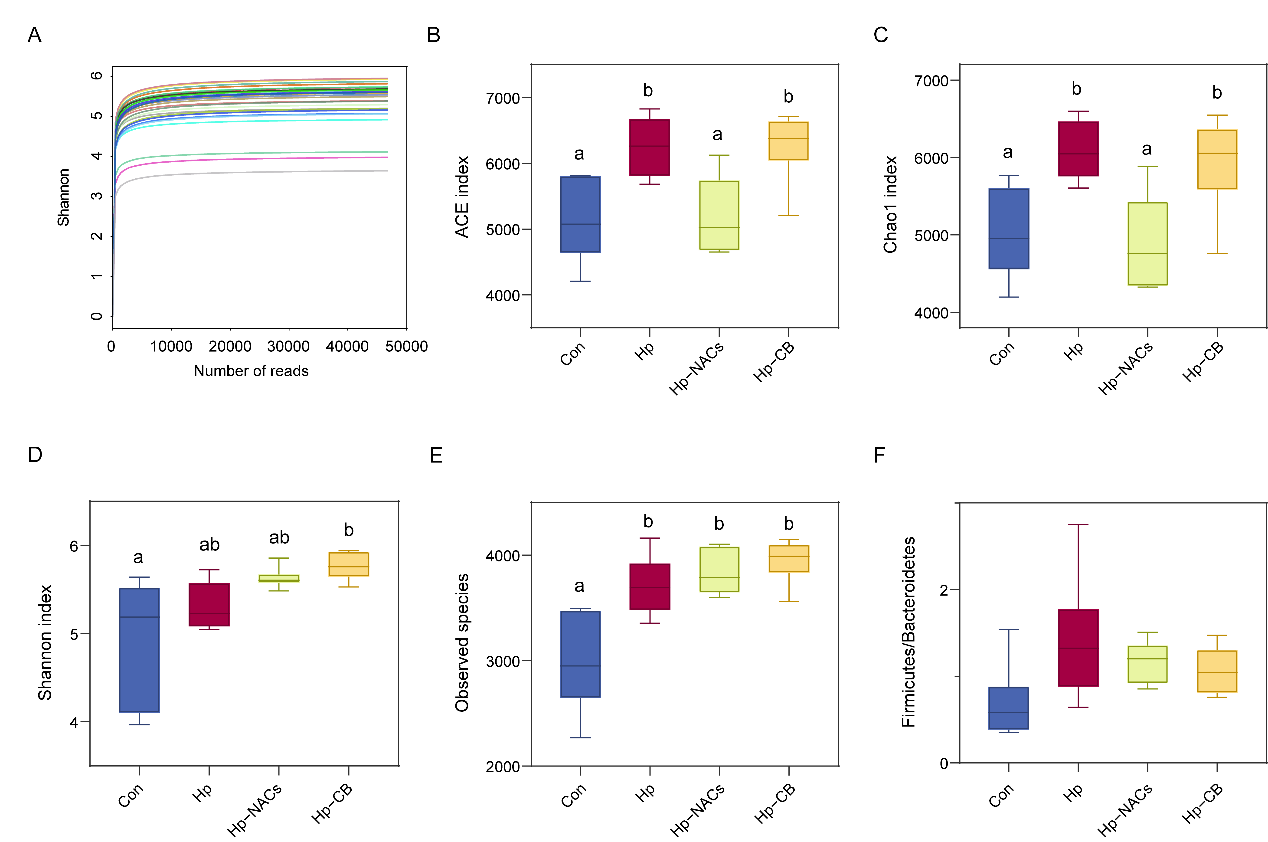


**Figure S2** Effect of *H. pylori* infection on the gut microbial community structure. (A) Rarefaction curves. (B) ACE index. (C) Chao1 index. (D) Shannon index. (E) Observed species. (F) The ratio of abundance between Firmicutes and Bacteroidetes. Bar plot is shown as means ± SD. Significance was evaluated using the 2-tailed, unpaired Student *t* test.
